# Maize Endosperm Development: Tissues, Cells, Molecular Regulation and Grain Quality Improvement

**DOI:** 10.3389/fpls.2022.852082

**Published:** 2022-03-07

**Authors:** Hao Wu, Philip W. Becraft, Joanne M. Dannenhoffer

**Affiliations:** ^1^Genetics, Development, and Cell Biology, Iowa State University, Ames, IA, United States; ^2^Department of Biology, Central Michigan University, Mount Pleasant, MI, United States

**Keywords:** kernel, differentiation, cell fate, genetics, seed physiology

## Abstract

Maize endosperm plays important roles in human diet, animal feed and industrial applications. Knowing the mechanisms that regulate maize endosperm development could facilitate the improvement of grain quality. This review provides a detailed account of maize endosperm development at the cellular and histological levels. It features the stages of early development as well as developmental patterns of the various individual tissues and cell types. It then covers molecular genetics, gene expression networks, and current understanding of key regulators as they affect the development of each tissue. The article then briefly considers key changes that have occurred in endosperm development during maize domestication. Finally, it considers prospects for how knowledge of the regulation of endosperm development could be utilized to enhance maize grain quality to improve agronomic performance, nutrition and economic value.

## Introduction

Cereal grains represent one of the key agricultural innovations upon which human civilization is founded. A distinguishing feature of cereal grains is their large, persistent, starch-filled endosperm that can be used directly as food or feed, ground to flour for many varied uses such as bread or pasta, malted and fermented for beverages or biofuel, or used as feedstock for industrial processes. Early peoples recognized particular grass seeds for their food value and began the process of domestication by selecting for traits that improved their value as crops. Grain size was an obvious trait that enhanced the caloric reward and ease of harvesting, and since endosperm constitutes the bulk of the grain, selection for larger grains inevitably increased endosperm size (Flint-Garcia, 2017).

From a developmental perspective, increasing grain size is a complex problem. From first principles, an increase in grain or endosperm size must entail either an increase in cell number or an increase in average cell size (or both). However, either of these changes could be accomplished by a variety of means: various alterations to the cell cycle or prolonged duration of active cell division. Any such changes require coordination amongst different tissues and cell types, as well as integration with the complex biochemical and physiological processes that occur during grain filling and seed maturation. Hence, selection pressure for grain traits during domestication and continued improvement either acts directly at the level of kernel and endosperm development, or affects changes in processes that then must be integrated into the developmental programs.

Understanding changes that occurred during domestication and improvement at the developmental and genetic level will greatly aid our ability to direct continued improvement and trait development. Methods are now available for identifying loci that were targets of selection as well as for introducing precise changes in the genome. Thus, it should now be possible to use genomic methods to help elucidate the developmental processes that were manipulated to provide the maize grains we have today, and to extend that knowledge into the future for continued improvement. Here we first describe the process of endosperm development from a kernel and cellular perspective, then review what is known of the molecular regulation of endosperm development, and finally we consider how this knowledge can be used for grain improvement.

## Kernel and Cellular Endosperm Development

### Endosperm Development in the Context of Whole Grain Development

**FIGURE 1:**
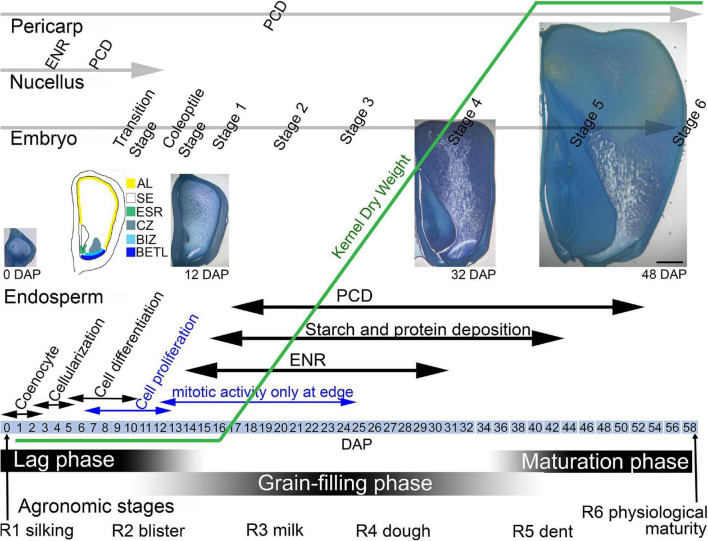
Maize kernel development from pollination to maturity highlighting endosperm growth and development in concert with phases of seed development and development of the embryo. AL, aleurone; BETL, basal endosperm transfer layer; BIZ, basal intermediate zone; CZ, conducting zone; DAP, days after pollination; ENR, endoreduplication; ESR, embryo surrounding region; PC, placenta-chalazal region; PCD, programed cell death; SA, subaleurone; SE, starchy endosperm.

The maize grain develops from a fertilized ovule to a mature kernel over the course of 50–60 days (Figure 1). The developing kernel contains tissues of maternal origin, the pericarp and nucellus, as well as those produced by double fertilization, the diploid embryo and the triploid endosperm. Agronomically, kernel development is described from the R1 silking stage to R6 physiological maturity but physiologically, kernel development is divided into the lag, grain-filling, and maturation phases (Egli, 2006; Abendroth et al., 2011). During the lag phase of growth, from 0 days after pollination (DAP) to as late as 15+ DAP, kernel dry weight gain is minimal as the endosperm and embryo develop, differentiate and increase in size. During the lag phase, the endosperm first undergoes free nuclear development, which involves mitotic divisions without cytokinesis, creating a multinucleate coenocyte (Olsen et al., 1995). Subsequent wall formation yields a completely cellular endosperm that further develops by an endosperm-wide proliferation of cells. By the end of the lag phase, mitotic activity becomes restricted to the peripheral layers of the endosperm (Sabelli and Larkins, 2009). Coincident with cell proliferation, four major cell types with specific functions differentiate within the endosperm: aleurone, basal endosperm transfer layer (BETL), embryo surrounding region (ESR) and starchy endosperm (SE) (Randolph, 1936; Kiesselbach, 1949; Leroux et al., 2014; Olsen, 2020). An additional 3–4 cell types develop later (Leroux et al., 2014). By the end of the lag phase, the endosperm accounts for about 60% of the kernel volume. During this phase, the embryo undergoes a formative division to produce the suspensor and embryo proper, then grows and transitions to a bilateral axis, and further develops to the coleoptile stage including establishment of shoot and root apical meristems (Sheridan and Clark, 2017). Also, during the early lag phase, the maternal nucellus tissue at first expands and accounts for a significant portion of the kernel but by ∼12 DAP it degenerates and remains only as the nucellar membrane (Randolph, 1936; Leroux et al., 2014). The exterior pericarp is also expanding and starts to develop thickened walls.

During the linear or grain-filling phase of seed development, (∼12–40 DAP), as endosperm cell proliferation slows, there is rapid water and weight gain as the SE cells expand with deposition of storage compounds and multiple rounds of endoreduplication (Sabelli and Larkins, 2009) (Figure 1). SE cells accumulate carbohydrates in the form of starch and seed storage proteins accumulate in protein bodies. Fully differentiated SE cells begin the process of programed cell death (PCD) (Young and Gallie, 2000; Dominguez and Cejudo, 2014). In the linear phase, the embryo continues growth and development and the pericarp cells begin to die. The final maturation stage of seed development involves PCD of all endosperm cells except the aleurone, final development of the embryo, and kernel desiccation and quiescence.

### Initial Endosperm Development and Cellularization

The development of the maize coenocyte begins with the first mitosis 2–5 h after fertilization and within 29 h of pollination (Randolph, 1936; Lowe and Nelson, 1946; Kiesselbach, 1949; Mol et al., 1994). The coenocyte is characterized by a large central vacuole surrounded by a thin layer of parietal cytoplasm. The duration of the coenocyte stage varies in maize lines and under different growth conditions but typically lasts for 2–3 days after pollination during which time nuclear divisions are synchronous (Randolph, 1936; Cooper, 1951; Kowles and Phillips, 1988; Leroux et al., 2014). Studies of genetic sectors indicate the first division identifies the sagittal left and right halves of the endosperm (McClintock, 1978) and this was later captured within a serially-sectioned endosperm (Monjardino et al., 2007). The second division occurs in the perpendicular plane specifying endosperm quarters, the third division specifies 8 longitudinal portions that describe conical sections of the endosperm. When there are only a few nuclei, they are located near the endosperm base but after several more rounds of division they migrate to occupy its full longitudinal extent (Randolph, 1936; Monjardino et al., 2007; Leroux et al., 2014). While no study has been undertaken to fully describe the cytoskeleton and its association with migrating nuclei, images of the maize coenocyte show nuclear cytoplasmic domains with radiating microtubules (Brown and Lemmon, 2007) reminiscent of those described for other cereals (Brown et al., 1996). The number of nuclei achieved during the coenocyte stage is 128–512 and it has been suggested that mitotic arrest at 256 or 512 nuclei is associated with commencement of cellularization (Olsen, 2001); however, Leroux et al. (2014) noted endosperm size not number of nuclei is coincident with cellularization.

Cellularization, which begins ∼ 3 days after pollination (DAP) and proceeds rapidly often being completed as early as 4 DAP, is coincident with the first size increase of the endosperm (Randolph, 1936; Cooper, 1951; Monjardino et al., 2007; Leroux et al., 2014). Anticlinal walls that are perpendicular to the outer endosperm wall form without mitosis to encase each parietal nucleus in a structure called an alveolus. Alveoli have walls on all sides of each nucleus except the face adjacent to the central vacuole. The first alveoli are formed at the base of the endosperm near the embryo, the region of future ESR (Randolph, 1936; Kiesselbach, 1949; Monjardino et al., 2007; Leroux et al., 2014). Within each alveolus, a mitotic division yields sister nuclei and a periclinal wall is deposited between them to produce an outer layer of cells and an inner layer of alveoli. Each alveolus has a successive series of anticlinal wall extension, nuclear mitosis, and periclinal wall deposition forming an externally positioned cell and an internally positioned new alveolus. Although cellularization is completed by successive alveolation in other cereals, maize appears to have a more random final partitioning of the central vacuole in the bulbous base of the endosperm, which may be related to the much larger size and unique shape of the maize endosperm (Leroux et al., 2014).

### Cell Proliferation and Cell Type Differentiation

Coenocyte development and cellularization sets up the basic body plan of the endosperm that then increases rapidly in size by cell proliferation and differentiates specialized cell types. During proliferation, endosperm growth occurs throughout the endosperm and is primarily associated with mitotic activity and increase in cell number generating the bulk of the endosperm (Kiesselbach, 1949; Kowles and Phillips, 1985). This contrasts with later stages when mitotic activity slows and becomes restricted to the kernel edge and endosperm growth is predominantly driven by cell and nuclear enlargement associated with storage deposition and endoreduplication (Randolph, 1936; Kiesselbach, 1949; Kowles and Phillips, 1985). Cell proliferation and cell type differentiation occur simultaneously and mitotic activity peaks ∼10 DAP (Kowles and Phillips, 1985). During the cell proliferation and differentiation period, the endosperm grows to exceed 60% of the kernel area (Leroux et al., 2014) and its shape has inverted with a large distal portion where cell divisions are still occurring and a narrower base where divisions have ceased, a shape which it will maintain for the rest of kernel development.

**FIGURE 2:**
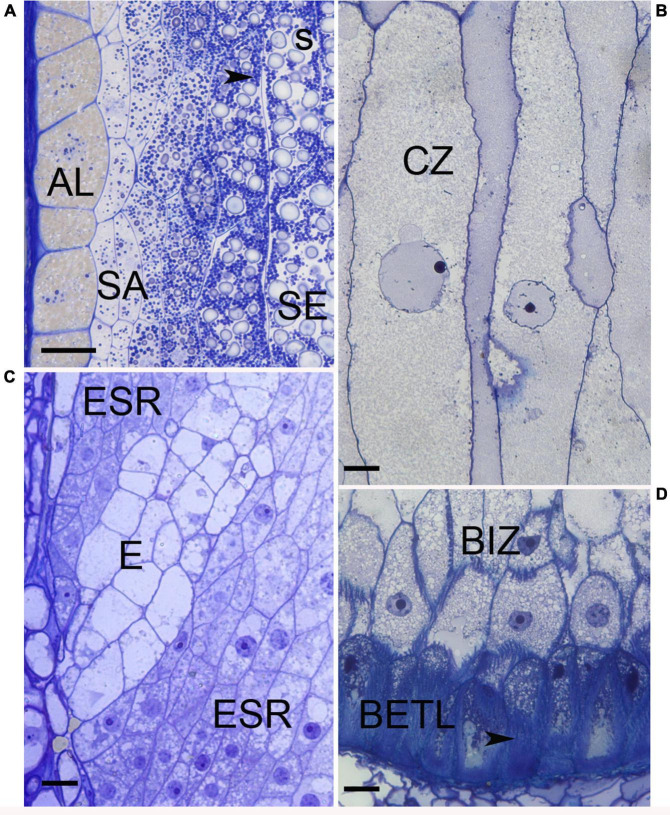
Light micrographs of maize cell types. **(A)** Edge of 20 DAP endosperm with aleurone (AL), subaleurone (SA), and starchy endosperm (SE). Within the SE abundant starch (s) and protein bodies (arrowhead) are present. **(B)** Conducting zone cells when first apparent, about 10 DAP. **(C)** Embryo surrounding region (ESR) cells at 8 DAP are restricted to surrounding the embryo suspensor (E). **(D)** Base of endosperm at 10 DAP when BETL cells have developed wall ingrowths (arrowhead) and adjacent basal intermediate zone cells (BIZ) are visible. Bars = 20 μm.

The larger maize endosperm differs from wheat, rice, and barley in that it develops a greater repertoire of specialized cell types (Olsen and Becraft, 2013). It is likely that breeding has impacted the development and functions of each of these cell types. The cell types are identified and described by a combination of their location, cell shape and contents, nuclear division patterns, wall elaborations, gene expression, and function (Table 1). The first cell types to become cytologically identifiable at 4-5 DAP are aleurone, BETL, ESR, and SE (Leroux et al., 2014). Several days later subaleurone (SA), conducting zone (CZ), and basal intermediate zone (BIZ) cells become distinguishable although some authors regard these as subtypes of SE and BETL (Becraft et al., 2001; Chourey and Hueros, 2017) (Figure 2). A last possible cell type, described as an endosperm region, is the endosperm adjacent to scutellum (EAS), only identifiable by location and transcriptome analysis because the cells have no identifiable cytological features that separate them from SE (Doll et al., 2020). Of these cell types, only AL and SE remain prominent at seed maturity.

**TABLE 1:** Maize endosperm cell type location, characteristics and function.

	Aleurone	BETL	ESR	SE	SA	CZ	BIZ	EAS
Location	Epidermal	Epidermal adjacent to placento-chalaza pad	Surrounds embryo early, later restricted to base near suspensor	The bulk of the endosperm tissue	Cell layer internal to aleurone, subtype of SE	In lower central portion of kernel, subtype SE	Between BETL and CZ, subtype BETL or SE	Adjacent to scutellum
Existence	4 DAP – seed maturity	4 DAP – completion grain fill	4–16 DAP	4 DAP – seed maturity	10 – ∼25 DAP cell division, seen at seed maturity	10–24 DAP	10- ? DAP	9–20 DAP
Size and shape	Small, cuboid	Elongate	Small, isodiametric	Irregular shape, very large	Small, cambial-like, wider than long	Very elongate, tapering ends	Elongate, prismatic	NA
Cytoplasm and wall	Prominent protein storage vacuoles, lipid bodies, thickened wall, in some genotypes anthocyanin	Apical end densely cytoplasmic, many mitochondria, Golgi extensive wall ingrowths, lignified wall	Densely cytoplasmic later becoming vacuolated; mitochondria, abundant rER	Vacuolated becoming filled with starch and protein bodies, enlarged up to 192C nuclei	Develop large protein bodies and small starch grains, high concentration of protein in this layer at seed maturity	Granular, non-distinct vacuoles, very large nuclei	Multiple vacuoles, moderately dense cytoplasm, wall ingrowths of flange type only	NA
Function	Storage lipids, proteins, minerals; remobilization of reserves for seedling growth	Transfer of solutes	Evidence for nutrient transfer, defense and signaling	Storage starch and proteins	Meristematic adding cells to edge, protein storage	Transport?	Radial distribution of solutes?	Transport between endosperm and embryo
Select references	Kyle and Styles, 1977; Reyes et al., 2011	Davis et al., 1990; Kang et al., 2009	Schel et al., 1984; Opsahl-Ferstad et al., 1997	Woo et al., 2001; Vilhar et al., 2002	Khoo and Wolf, 1970; Lending and Larkins, 1989	Zheng et al., 2014	Davis et al., 1990; Monjardino et al., 2013	Doll et al., 2020

*Aleurone* and BETL are a single cell layer that is initiated by the first periclinal wall deposition at the beginning of the cellularization process and are in essence the epidermal layer of the endosperm tissue (Figure 1). Aleurone cells cover the surface except for at the base of the endosperm adjacent to the maternal placenta-chalazal region (PC) where the BETL and ESR cells are located. After the first division, the nascent aleurone cells have microtubular preprophase bands that mark the future plane of cell division whereas the underlying cells do not (Brown and Lemmon, 2007). At first, cell divisions are at right angles to each other to maintain a cuboid shape, but later divisions occur predominantly in anticlinal planes so surface expansion can keep pace with increasing kernel volume. When periclinal divisions occur, the inner cell switches from aleurone into SE and positioning rather than lineage has been shown to be important for aleurone cell specification (Becraft and Asuncion-Crabb, 2000; Gruis et al., 2006). Cytologically, aleurone cells become apparent by 5 DAP with development of multiple large vacuoles (Kyle and Styles, 1977). Between 10–15 DAP the cells become more distinct as aleurone by beginning to accumulate protein and membranes within smaller vacuoles, forming abundant lipid bodies, and developing a thickened wall (Kyle and Styles, 1977; Reyes et al., 2011). Protein eventually occupies a large proportion of the vacuoles and they develop the characteristic ultrastructure of aleurone bodies or protein storage vacuoles (PSV). These PSV contain many internal structures including a large protein inclusion containing zeins, α-globulin and legumin-1. The zeins in the vacuole apparently transport from the ER to prevacuolar compartments then to PSVs by an atypical autophagic process (Reyes et al., 2011). PSVs also contain phytic acid crystals, a glycoprotein containing matrix, and intravacuolar membranes (Reyes et al., 2011). Late in development, abundant lipid bodies surround the PSVs and the aleurone cell wall thickens considerably (Kyle and Styles, 1977). The colorful kernels that characterize many maize genotypes are produced by anthocyanin deposition in aleurone cells (or sometimes the pericarp). Aleurone cells typically do not endoreduplicate and they are the only endosperm cells that do not enter PCD during development (Burton and Fincher, 2014); thus, they are the only living endosperm cells at seed maturity. Aleurone cells function in mineral, lipid, and protein storage. At germination the aleurone secretes hydrolytic enzymes that digest the starches and proteins stored in the SE and release sugars and amino acids for use by the growing seedling.

*Basal endosperm transfer layer* cells are epidermal cells that form an interface between the basal filial tissues and the maternal pedicel tissue (Figure 2). With elaborate wall ingrowths that increase the surface area of the plasma membrane, they typify transfer cells that are important in solute movement across the apoplastic interface from the maternal tissues into the growing grain where they can be used for growth or assimilated into storage compounds (Zheng and Wang, 2010; McCurdy, 2015). Various authors describe the inner several (2–3) cell layers with similar but distinct cytological features as an extension or inner part of the BETL (Kiesselbach and Walker, 1952; Davis et al., 1990; Gao et al., 1998; Monjardino et al., 2013). However, the distinct cytological characteristics and specific gene expression within these inner cells (Leroux et al., 2014; Li et al., 2014) was used to segregate them from the BETL as the basal intermediate zone (BIZ). Future work is needed to fully describe the characteristics and occurrence of the BIZ and CZ cells to understand their identities, locations, and relationships to the well-known adjacent BETL and SE. Here we will describe the BETL using the features of the most basal cell layer.

BETL cells begin elongation and wall ingrowth deposition ∼6 DAP (Kiesselbach and Walker, 1952; Charlton et al., 1995; Kang et al., 2009; Monjardino et al., 2013; Leroux et al., 2014). Before basal wall ingrowth begins there is already a polarized distribution of mitochondria with many of them located adjacent to the lower wall (Kang et al., 2009). As the plasma membrane invaginates and ingrowths begin to form, there is an increase in mitochondria, Golgi, and *trans*-Golgi-network vesicles indicating high secretory activity (Davis et al., 1990; Charlton et al., 1995; Kang et al., 2009; Monjardino et al., 2013). Within the kernel, there is a gradient of development of BETL cells from near the embryo to the abgerminal side of the endosperm (Hueros et al., 1999).

Developing cells take on the full complement of cytological features after ∼12–16 DAP with an asymmetric distribution of nucleus and dense cytoplasm at the distal end above prominent wall ingrowths adjacent to the PC (Kiesselbach and Walker, 1952; Davis et al., 1990; Kang et al., 2009). The cytoplasm contains variously shaped nuclei, numerous mitochondria, Golgi, and enlarged vesicles; whereas, cytoplasm interstices among the wall ingrowths are packed with ER and abundant mitochondria (Davis et al., 1990; Kang et al., 2009). Plasmodesmata are located in the primary cell wall in between ingrowths and interconnect BETL cells but are absent between BETL cells and the underlying PC (Davis et al., 1990; Monjardino et al., 2013). The wall ingrowths have a complex architecture with abundant parallel, rib-like projections called flange ingrowths that anastomose along their length (Davis et al., 1990; Talbot et al., 2002; Monjardino et al., 2013; McCurdy, 2015). In the lower region of the cells, these flange wall ingrowths are interconnected by lateral extensions so wall material more or less fills the cell volume (Davis et al., 1990; Talbot et al., 2002; Kang et al., 2009). The interconnecting wall architecture is equated to reticulate wall ingrowths (Monjardino et al., 2013); although others view them as an elaboration of the flange outgrowth (Talbot et al., 2002; McCurdy, 2015). Regardless of their origin, this secondary interconnection of flange ingrowths only exists in the first cell layer BETL and not BIZ cells. Using a variety of detection methods, lignin was found in BETL cell walls within both types of wall ingrowth (Rocha et al., 2014).

*Basal intermediate zone* cells become apparent later than the BETL about the same time as the adjacent CZ, around 8–10 DAP (Kiesselbach and Walker, 1952; Leroux et al., 2014). Cells are more elongate than the adjacent BETL and have oblique ends, sometimes being described as prismatic (Figure 2). They have moderately dense cytoplasm, abundant vesicles, and nuclei slightly larger than the adjacent BETL (Kiesselbach and Walker, 1952; Davis et al., 1990; Leroux et al., 2014). Flange wall ingrowths get progressively shorter and fewer in number within the cells adjacent to the CZ (Davis et al., 1990; Kang et al., 2009; Monjardino et al., 2013). Adjacent cells often have the wall ingrowths at the same location giving cross sections of the cells a distinctive appearance. The walls in between the ingrowths have many plasmodesmata these being most abundant in cells near the CZ, (Davis et al., 1990; Gao et al., 1998; Monjardino et al., 2013). It has been suggested that cell wall features, ingrowths and abundant plasmodesmata, might serve the function of radial distribution of solutes (Davis et al., 1990).

*Embryo surrounding region* cells are the earliest endosperm cells to differentiate, as early as 4 DAP and can even be apparent as the last of the central vacuole becomes cellular. They are located adjacent to the embryo and completely encircle the embryo at first but by 7 DAP they only form a semi-circle of cells located around the suspensor of the embryo (Figure 2) (Schel et al., 1984; Opsahl-Ferstad et al., 1997). ESR cells are small, isodiametric, and have dense cytoplasm with small vacuoles. Cytoplasmic characteristics that suggest high metabolic activity include: large nuclear to cell volume, prominent mitochondria, an abundant network of rER with large intracisternal spaces and Golgi associated with many vesicles. Around 15 DAP, ESR cells are the first cells in the endosperm to undergo PCD as the embryo expands (Dominguez and Cejudo, 2014). Proposed functions of the poorly understood ESR include, pathogen defense, embryo/endosperm signaling, nutrient transport from endosperm to embryo and perhaps protecting the embryo from fluxes of auxin (Cossegal et al., 2007; Chen et al., 2014).

*Starchy endosperm* functions as the major nutrient storage site in the endosperm and occupies the greatest portion (by volume or weight) of the kernel. SE cells differentiate from the inner cells produced by the cellularization process and new cells formed by peripheral divisions in the aleurone/subaleurone layers (Figure 2). Accordingly, cell differentiation, endoreduplication and storage deposition within them occurs in a developmental pattern from the kernel crown to base and the center to the edge with the oldest, largest, and most developed SE cells in the endosperm center (Kiesselbach, 1949; Kowles and Phillips, 1988). Beginning ∼10 DAP as SE cells enlarge, they cease mitosis and undergo multiple rounds of endoreduplication that increase the DNA content and nuclear size (Kowles and Phillips, 1985). It is at this time that the cells begin to accumulate starch and storage proteins. Endoreduplication, characterized by DNA replication without chromatid separation, yields multiple copies of the nuclear DNA and 4–5 cycles of endoreduplication (up to 192C) is common (Sabelli and Larkins, 2009; Dante et al., 2014). There is a positive relationship between *C*-value, nuclear size and cell size and in 16 DAP endosperm, the centrally-located largest cells, with the highest *C*-values, were fewest in number (less than 7% of the total cells) but accounted for 60% of the endosperm volume (Vilhar et al., 2002). Starch granules that account for ∼70% of the final kernel dry weight are semi-crystalline structures synthesized from densely-packed amylose and amylopectin (Hannah, 2007). Protein storage in the SE cell involves both the prolamin zeins, which are synthesized and accumulate in rER derived protein bodies, and globulins that accumulate in PSV (Khoo and Wolf, 1970; Lending and Larkins, 1989; Woo et al., 2001; Arcalis et al., 2010). Zeins account for over 60% of the kernel protein and are high in proline and glutamine but low in several essential amino acids (lysine, methionine, and tryptophan) significantly affecting the nutritional value of seeds. Zeins fall within 4 classes and the presence and abundance of different classes within protein bodies affects protein body size and association with the starch granules affecting physical characteristics of kernel (Holding, 2014). The nascent protein body forms with deposition of γ-zeins within small protein bodies. In larger, more mature protein bodies, γ- and β-zeins localize to a peripheral position after abundant α-zein and δ-zein is deposited in the internal portion (Lending and Larkins, 1989; Woo et al., 2001; Guo et al., 2013). Gene expression patterns of developing endosperm shows γ- and β-zeins transcripts occur throughout the endosperm at 10 DAP whereas α-zein transcripts are confined to the germinal edge reiterating the central to edge pattern of kernel development (Woo et al., 2001). Endoreduplication and storage deposition continue until the cells undergo PCD, which begins in central SE cells about 16 DAP (Young et al., 1997).

*Subaleurone* cells are located just inside the aleurone in all portions of the endosperm except the base near the BETL. Early workers described the area as cambial-like because periclinal divisions generate linear files of cells that contribute the last cells to the edge of the SE (Randolph, 1936; Kiesselbach, 1949; Cooper, 1951). Subaleurone cells are small in size and the cytoplasm is characterized by numerous mitochondria, proplastids, some aleurone-like lipid bodies and ER but little starch and few protein bodies are present (Khoo and Wolf, 1970; Lending and Larkins, 1989). In mature kernels, the subaleurone is still distinct from the adjacent SE as the cells are smaller, the protein bodies are larger, and the cells lack large starch grains (Duvick, 1961; Khoo and Wolf, 1970).

*Endosperm adjacent to scutellum* may be a new cell type defined by location and transcriptome but the cells are cytologically indistinguishable from the adjacent SE (Doll et al., 2020). EAS is first detectable at 9 DAP as 2–3 layers of cells whose transcriptome has an enrichment of transporter genes compared to the rest of the endosperm. This area is identifiable until about 20 DAP and presumably facilitates nutrient supply or communication across the endosperm-embryo interface.

*Conducting zone* cells have received little cytological study and descriptions are all very brief and appear to include BIZ, BETL, and/or SE (Kiesselbach and Walker, 1952; Davis et al., 1990; Monjardino et al., 2013). Several authors have noted a core of elongate cells within the base of the endosperm extending above the BETL and suggested they were vascular-like and had a conducting function (Brink and Cooper, 1947; Cooper, 1951; Charlton et al., 1995). Recently, several studies have more completely described the CZ as a distinct cell type apart from BETL and BIZ cells (Leroux et al., 2014; Zheng et al., 2014). The cells are extremely elongate with tapering end walls and are distinct from neighboring cells by their much larger size, granular cytoplasm, prominent enlarged nuclei, few starch grains, lack of wall ingrowths, and gene expression profiles. At ∼ 24 DAP, these cells are thought to begin to degenerate.

## Molecular and Genetic Regulation of Endosperm Development

Although the endosperm is composed of several distinct tissues and multiple cell types, each of which contributes uniquely to the biology of the grain, the majority of molecular work has focused on understanding the BETL, aleurone, SE and the ESR, which will each be considered here.

### Regulation of Basal Endosperm Transfer Layer Cell Development

The first cells to form during endosperm cellularization give rise to the BETL and aleurone, however, evidence suggests that BETL specification may begin earlier, perhaps in the megagametophyte (Gutierrez-Marcos et al., 2006). The *baseless1* (*bsl1*) mutant causes patterning defects in the embryo sac, which subsequently manifest during endosperm development as disorganized BETL, including mispatterned basal gene expression. Furthermore, several gene transcripts show basal-specific accumulation beginning in coenocytic endosperm prior to cellularization. This suggests that BETL specification begins prior to endosperm cellularization and maybe in the embryo sac before fertilization. Following cellularization, the transfer cells differentiate and form a morphological gradient along the basal-apical axis, grading into the BIZ and CZ, and along the germinal-abgerminal axis (Gómez et al., 2009). As discussed below, this pattern might be associated with concentration gradients of inducing factors such as hormones or sugars.

#### Myb Related Protein1 Is a Central Regulator of Basal Endosperm Transfer Layer Cell Fate

MRP1 is a transcription factor (TF) and a determinant of BETL cell fate because ectopic expression of *MRP1* was sufficient to cause early aleurone cells to acquire BETL identity (Gómez et al., 2009). MRP1 is specifically expressed in the BETL and directly activates expression of several other BETL-specific genes, collectively known as *basal endosperm transfer layer* (*betl*) genes (Gómez et al., 2002; Barrero et al., 2006). Several *betl* genes encode peptides that are secreted into surrounding pedicel tissue and have antifungal properties suggesting they are protective for the seed (Cai et al., 2002).

A transcriptomic analysis identified *MRP1* as a hub gene for the BETL compartment and among the genes in the expression module were six additional BETL-specific TFs, which may in turn regulate additional downstream BETL-specific genes (Zhan et al., 2015). Other MRP1 targets of particular note include *maternally expressed gene-1* (*meg1*) and two cytokinin response regulator (RR) genes, *ZmTCRR1* and *ZmTCRR2* (Gutiérrez-Marcos et al., 2004; Muniz et al., 2006, 2010; Gómez et al., 2009).

#### *MEG1* and Imprinting Control Basal Endosperm Transfer Layer Development

*MEG1* is expressed specifically in the BETL and encodes a small cysteine-rich secreted peptide proposed to function as a developmental signaling molecule (Gutiérrez-Marcos et al., 2004; Costa et al., 2012). Indeed, *meg1* RNAi caused a severe reduction in BETL development in the basal endosperm, while ectopic expression of *MEG1* also caused ectopic BETL formation; hence, MEG1 is necessary and sufficient for BETL differentiation (Costa et al., 2012). These treatments were accompanied with a corresponding reduced expression, or ectopic expression of *MRP1*, respectively. Thus, *MEG1* and *MRP1* both act as determinants of BETL fate and their expression is regulated by a feedback loop of mutual reinforcement.

*MEG1* is an imprinted gene where maternally inherited copies are expressed while the paternal allele is silenced by DNA methylation (Gutiérrez-Marcos et al., 2004). Imprinting is often hypothesized as being involved in controlling resource allocation (Rodrigues and Zilberman, 2015). To test this hypothesis, a synthetic *Meg1* gene (*synMeg1*) was constructed where codon replacement produced a coding region with little nucleotide sequence similarity to the endogenous gene. This was then placed under the control of the *bet9* promoter, which is BETL-specific but is not imprinted. Transgenic maize showed a dosage dependent increase in the extent of BETL cell differentiation and a concomitant increase in kernel size and weight, whereas, *synMeg1* under the control of the native (imprinted) *Meg1* promoter did not produce this effect (Costa et al., 2012). These results are consistent with imprinting of *Meg1* regulating resource acquisition in the kernel by limiting BETL development.

#### Hormone Signaling and Basal Endosperm Transfer Layer Development

Several lines of evidence suggest that cytokinin (CK) phytohormone may be important for BETL development and/or function. CKs are often associated with sink formation and they accumulate in developing maize kernels, peaking during the period of maximum grain filling. The accumulation pattern mirrors expression of *ZmIPT2*, which encodes isopentenyltransferase, a CK biosynthetic enzyme. *ZmIPT2* is most strongly expressed in the BETL, where the highest CK levels occur (Brugiere et al., 2008).

The CKs signal *via* a pathway where histidine kinase (HK) receptors signal *via* histidine phospho-transfer protein (HP) to regulate the activity of response regulators (RRs) that control expression of downstream factors (To et al., 2008). There are two types of RRs; type-B RRs are MYB family TFs that function as positive effectors of CK signaling, whereas, type-A RRs are negative regulators of CK signaling. In response to CK, HPs phosphorylate type–B RRs, which activates them and allows them to control CK-regulated gene expression. One type–B RR target is activation of type-A RR expression. A pair of type-A RR genes, *ZmTCRR1* (*Zea mays Transfer Cell Response Regulator1*) and *ZmTCRR2*, are specifically expressed in the BETL (Muniz et al., 2006, 2010). Gene expression is highest during the period of maximum BETL differentiation suggesting a possible role in directing BETL development. The protein products of these genes are not restricted to BETL cells but accumulate along a concentration gradient into the CZ, coincident with the gradient in cell morphology.

It remains unclear whether these RR-like genes are in fact involved with CK signaling. There is no experimental evidence for CK regulation and the TCRRs contain some sequence features not found in bona fide RRs (Muniz et al., 2010). As mentioned, *ZmTCRR1* and *ZmTCRR2* are direct targets of regulation by MRP1. Intriguingly, MRP1 contains a motif similar to the GARP motif found in type-B RRs, suggesting either that MRP1 is a type-B RR performing an as yet undiscovered role in CK signaling, or that this represents a regulatory system descended from canonical RRs but no longer connected to hormone signaling.

#### Sugar Induction of Basal Endosperm Transfer Layer Differentiation

One of the major functions of the BETL is sugar transport and interestingly, several studies have revealed that sugar plays an essential role in promoting BETL differentiation. Proteins involved in sugar import include SWEET sugar transporters and invertase, which cleaves sucrose to glucose and fructose. The *miniature1* (*mn1*) gene is a BETL-specific gene that encodes a cell wall invertase, and *ZmSWEET4c* is also a BETL-specific gene encoding a plasma membrane hexose transporter (Sosso et al., 2015). Mutations in both genes impair sugar transport into developing kernels causing substantial decreases in grain size (Miller and Chourey, 1992; Cheng et al., 1996; Kang et al., 2009; Sosso et al., 2015). Notably, mutants in both genes show dramatically decreased BETL formation showing that sugar flux is required to promote BETL cell differentiation. Furthermore, the expression of *ZmSWEET4c* and *mn1*, as well as *MRP1*, are all sugar inducible, particularly with glucose, and expression of all these genes is decreased in the *zmsweet4c* mutant (Barrero et al., 2009; Sosso et al., 2015). What emerges is a feed-forward model where sugar induces the expression of sugar transport machinery as well as BETL differentiation, which then facilitates increased sugar transport into the endosperm and further reinforces this system (Sosso et al., 2015).

Transport of other classes of molecules is also critical; the choline transporter-like protein1 (ZmCTLP1) is encoded by the *small kernel 10* (*smk10*) gene. Mutations of *smk10* disrupt BETL differentiation, decrease kernel size, and cause extensive alterations in endosperm lipid composition and content (Hu et al., 2021).

### Regulation of Aleurone Development

Aleurone is a metastable cell type requiring continuous positional cues to establish and perpetuate aleurone identity (Becraft and Asuncion-Crabb, 2000). Genetic studies identified several factors that are likely involved in signaling aleurone fate. Mutants of *defective kernel 1* (*dek1*) lack aleurone, showing it is essential for aleurone development (Becraft and Asuncion-Crabb, 2000; Becraft et al., 2002; Lid et al., 2002). DEK1 is a plasma membrane-localized protein consisting of several domains. An intracellular region includes a calpain-family proteinase domain whose cysteine protease activity is stimulated by Ca^++^ (Wang et al., 2003; Tian et al., 2007). A transmembrane domain contains a series of 21–24 membrane-spanning helices, depending on the molecular prediction, interrupted by a “loop” that is either cytoplasmic or extracellular, depending on the model (Lid et al., 2002; Kumar et al., 2010). This transmembrane domain is required for mechanosensitive Ca^++^ channel activity, leading to the hypothesis that tension on epidermal (aleurone) cells may trigger Ca^++^ (Tran et al., 2017). This in turn would activate the proteinase and regulate aleurone cell fate through cleavage of yet unidentified signal transduction substrates.

CRINKLY4 (CR4) is a plasma membrane receptor-like kinase that is likewise involved in promoting aleurone fate through an unknown signaling mechanism (Becraft et al., 1996; Jin et al., 2000). Mutants of *cr4* impair aleurone development often producing mosaic kernels partially lacking aleurone (Becraft et al., 1996; Becraft and Asuncion-Crabb, 2000). Genetic evidence suggests that the DEK1 and CR4 signaling pathways converge although the molecular details remain obscure (Becraft et al., 2002).

Whereas most wild type maize lines contain just a single layer of aleurone cells, mutants in the *supernumerary aleurone 1* (*sal1*) gene produce multiple aleurone layers, indicating SAL1 is normally required to restrict aleurone formation (Shen et al., 2003). SAL1 is a class E vacuolar sorting protein, related to human CHMP1, involved in internalization and sorting of plasma membrane proteins into multivesicular bodies for degradation (Spitzer et al., 2009). The SAL1, DEK1 and CR4 proteins colocalized in endosomes suggesting that SAL1 may limit DEK1 and CR4 levels by protein degradation (Tian et al., 2007). This was hypothesized to limit levels of DEK1 and CR4 signaling and thus restrict the number of aleurone layers.

The *thick aleurone1* (*thk1*) mutation also causes multiple aleurone cell layers indicating THK1 is another negative regulator of maize aleurone cell fate (Becraft and Yi, 2011). The *thk1* gene encodes a homolog of NEGATIVE ON TATA-LESS1 (NOT1), a protein that acts as a scaffold for the CARBON CATABOLITE REPRESSION4-NEGATIVE ON TATA-LESS (CCR4-NOT) complex that mediates many mRNA-related processes, including RNA-turnover, transcription initiation and elongation, translation, and RNA quality control (Wu et al., 2020). The mutant of *thk1* alters expression of genes associated with cell division, cell communication, hormone response, and plant epidermis development, which may contribute to generating multiple aleurone cell layers (Wu et al., 2020). Interestingly, the *thk1* mutant is epistatic to *dek1*; double mutants showed multiple aleurone layers like *thk1*, even though *dek1* mutants are unable to form aleurone, indicating THK1 is a likely component of the signaling system downstream of DEK1 (Becraft and Yi, 2011).

The *naked endosperm* (*nkd*) mutant is a duplicate factor that disrupts aleurone differentiation, producing endosperm with multiple layers of peripheral cells only partially differentiated as aleurone (Becraft and Asuncion-Crabb, 2000). The corresponding genes, *nkd1* and *nkd2*, encode INDETERMINATE DOMAIN (IDD) family C2H2 zinc finger TFs, ZmIDDveg9 (NKD1) and ZmIDD9 (NKD2), respectively (Colasanti et al., 2006; Yi et al., 2015). The NKD1,2 TFs regulate genes important for several aspects of aleurone function including, cell growth and division, anthocyanin accumulation, lipid storage, pathogen defense and abscisic acid (ABA) response (Gontarek et al., 2016; Gontarek and Becraft, 2017).

NKD1,2 and THK1 may co-regulate aleurone cell development. Triple mutants of *nkd1;nkd2;thk1* reveal an additive relationship between *nkd1,2* and *thk1* on controlling the number of aleurone cell layers, and an epistatic relationship on aleurone cell differentiation (*nkd1,2* is epistatic to *thk1*); triple mutants have many layers of partially differentiated aleurone (Becraft and Yi, 2011). This indicates that NKD1,2 and THK1 may negatively regulate aleurone cell fate through independent pathways, and that NKD1,2 is required for aleurone cell differentiation downstream of THK1. A co-expression network analysis between *nkd1,2* and *thk1* mutants suggests that NKD1,2 and THK1 may co-regulate cell cycle and division to restrict aleurone development to a single cell layer, whereas NKD1,2, but not THK1, may regulate auxin signaling to maintain normal aleurone differentiation (Wu and Becraft, 2021).

A recent study suggests that adequate iron content is critical for proper aleurone development (He et al., 2021). The *shrunken4* (*sh4*) gene of maize encodes a YELLOW STRIPE-LIKE2 (ZmYSL2) metal transporter that controls iron abundance in aleurone cells. The *sh4* mutant causes loss of aleurone cell identity accompanied with decreased iron accumulation. The basis for this requirement is unknown but it seems likely that iron may be an essential cofactor for one or more components of the aleurone cell fate machinery. Interestingly, the GO term “iron ion binding” was over-represented among DEGs of *nkd* mutant aleurone (Gontarek et al., 2016).

### Regulation of the Embryo Surrounding Region

The ESR is a poorly understood tissue and little is known about how its development is regulated. Weighted gene coexpression network analysis (WGCNA) identified an ESR-specific gene coexpression module, which was enriched for genes involved in cell–cell signaling, consistent with the proposed function of these cells in signaling between the endosperm and embryo (Zhan et al., 2015). ESR-specific promoters were identified and several putative *cis* elements were identified, but most appear to be shared amongst other endosperm cell types (Bonello et al., 2000; Zhan et al., 2015). Several TFs were identified with high module membership scores for the ESR-specific module, which are good candidates for testing experimentally (Zhan et al., 2015).

No bona fide ESR mutant has yet been reported. This could be because disruption of the ESR is lethal, because such mutants are too subtle, or because of genetic redundancy. The *shohai* (*shai*) mutant disrupts the formation of the embryo pocket, a cavity in the endosperm that is normally filled by the embryo (Mimura et al., 2018). In non-concordant kernels, a *Shai* wildtype endosperm rescued the development of mutant embryos whereas, wildtype embryos caused the formation of a normal embryo pocket in mutant endosperms. Thus, SHAI is involved in signaling between the endosperm and embryo, and as a TF of the RWP-RK family, is a good candidate for controlling ESR development. To date, a detailed analysis of the ESR has not been reported for this mutant.

### Molecular Regulation of Starchy Endosperm Development

The SE is the most economically valuable part of a grain and, as such, considerable effort has been expended to understand the molecular and biochemical regulation of SE development, particularly as it pertains to protein and starch accumulation. Yet, despite this attention, little is known about the regulation of SE cell fate. Among TFs that regulate SE development, OPAQUE-2 (O2) is a basic leucine zipper (bZIP) TF that has long been recognized as key to regulating the biosynthesis and accumulation of nutrient materials in SE. First shown to activate the expression of the 22-kD α-zein gene (Schmidt et al., 1990, 1992), a genome-wide transcriptional regulatory network study showed that O2 also regulates several additional zein storage protein genes, genes for carbon fixation (PPDK1 and PPDK2), and additional downstream transcription factors (GBF and Myb-like TFs) (Li et al., 2015). Further, proteomic studies showed that protein levels of Granule-Bound Starch Synthase I (GBSSI), Starch Synthase IIa (SSIIa), and Starch Branching Enzyme I (SBEI) were reduced in *o2* mutants, indicating that O2 may also play an important role in starch biosynthesis, albeit indirectly (Jia et al., 2013; Zhang et al., 2016).

PROLAMIN-BOX BINDING FACTOR1 (PBF1) is a TF that can bind to the *prolamin-box* in promoters of the 27-, 22, and 19-kD zein genes (Wang et al., 1998). Moreover, starch content was reduced in *pbf1* RNAi knockdown mutants, suggesting that PBF1 also affects starch accumulation (Zhang et al., 2016; Qi et al., 2017). Compared with the *o2* or *pbfRNAi* single mutants, the *o2*; *pbfRNAi* double mutant caused further reduction of zein protein and starch content suggesting that O2 and PBF function additively and synergistically to regulate gene networks in SE development (Zhang et al., 2015, 2016).

In addition to O2-PBF1 interactions, O2 also interacts with other TFs, including O2 HETERODIMERIZING PROTEINS (OHPs), ZmbZIP22 and ZmMADS47, forming a complex that regulates expression of zein genes (Li and Song, 2020; Dai et al., 2021). Two NAC transcription factors, ZmNAC128 and 130, are also involved in regulating zein and starch biosynthetic genes (Zhang et al., 2019).

NKD1,2 are involved in SE development in addition to their roles in aleurone formation. Laser-capture microdissection RNA sequencing (LCM RNAseq) showed the *nkd1,2* genes are expressed in both aleurone and SE. The *nkd1,2* mutants have an opaque, floury endosperm phenotype accompanied by widespread changes in expression of genes regulating starch biosynthesis, storage proteins or other cellular components (Yi et al., 2015; Gontarek et al., 2016).

In addition to nutrient biosynthesis and accumulation, endoreduplication and PCD are important features of SE development. As the endosperm transitions from the cell division to nutrient accumulation phases, the cell cycle transitions from mitotic cell division to endoreduplication (Larkins et al., 2001). Cyclin-dependent Kinases (CDKs) are key regulatory factors affecting endoreduplication, among which A-type CDK (CDKA) functions in S-phase and B-type CDK (CDKB) functions to promote G2 to M phase transition. Endoreduplication may be triggered by induction of CDKA and inhibition of CDKB (Grafi and Larkins, 1995; Sabelli, 2012). RETINOBLASTOMA-RELATED (RBR) proteins and CDK inhibitor (CKI) proteins are additional cell cycle regulators that may control the transition to endoreduplication. An *rbr1* mutant with decreased expression resulted in enhanced endoreduplication suggesting RBR1 is a negative regulator (Sabelli et al., 2013). Two different families of CKIs have been implicated to regulate endoreduplication, Kip-related proteins (KRPs) and SIAMESE (SIA) proteins (Coelho et al., 2005; Zhang et al., 2020). Members of both families are expressed at appropriate times in SE and overexpression of KRP1 in maize callus promoted endoreduplication. Hormones also appear to influence endoreduplication, with auxin promoting the initiation and maintenance of endoreduplication, whereas, cytokinin inhibited proliferating cells from entering the endoreduplication cycle (Sabelli, 2012).

Beginning at around 12-16 DAP, SE cells undergo PCD (Young and Gallie, 2000) with the initiation of PCD promoted by ethylene and sugar accumulation (Bhave et al., 1990; Young et al., 1997). The maize ABA-insensitive *viviparous1* (*vp1*) mutant showed increased levels of ethylene and accelerated progression of PCD, indicating that ABA might inhibit PCD *via* negatively regulating ethylene synthesis (Young and Gallie, 2000). Cell cycle regulation also interfaces with PCD as the *rbr1* mutant enhanced PCD, suggesting negative effects of RBR1 on both PCD and endoreduplication in maize SE (Sabelli, 2012). The details of the connections among hormones, cell cycle control factors, endoreduplication, and PCD are still unclear and require further characterization.

### Transcription Networks and Endosperm Development

Different endosperm cell types have distinct transcriptomes and recent studies have begun to unravel the gene regulatory networks (GRNs) that underlie endosperm development and function (Zhan et al., 2015; Gontarek et al., 2016; Zhang et al., 2016; Feng et al., 2018; Ji et al., 2021; Wu and Becraft, 2021). Some of the key TFs known to regulate specific processes during endosperm development were discussed above. In addition, there are extensive inter-regulatory relationships among many of these TFs. These involve direct regulation of target genes as well as indirect regulation *via* downstream TFs. NKD1 and NKD2 directly regulate each other’s expression as well as directly promote expression of other important TFs including O2 and PBF1 (Gontarek et al., 2016). The DOF3 TF gene is also regulated by NKD1 and NKD2 but most likely indirectly. DOF3 in turn directly regulates the expression of NKD1 and NKD2 (Qi et al., 2017) while O2 promotes expression of NKD2 (Zhan et al., 2018). NKD2 is also regulated by OPAQUE11 (O11, a bHLH TF), which also regulates the expression of DOF3, O2, and PBF (Feng et al., 2018). O2 also transactivates ZmGRAS11, a GRAS-family transcription factor without a DELLA domain, which then directly regulates the expression of ZmEXPB12, a cell wall loosening protein important for cell expansion (Ji et al., 2021). This suggests that O2 not only regulates grain filling, but also controls cell expansion in developing endosperm. ZmABI19 is a B3 domain TF that regulates expression of multiple key TF genes, including O2, PBF1, ZmbZIP22, NAC130, and O11, indicating an important role in maize seed development and grain filling (Yang et al., 2021). Furthermore, a recent study proposed a model for O2 nuclear translocation mediated by the SnRK1-ZmRFWD3 pathway (Li et al., 2020). At high sucrose levels, the sucrose-responsive protein kinase SnRK1 phosphorylates ZmRFWD3, an E3 ubiquitin ligase, leading to ZmRFWD3 degradation. At low sucrose levels, SnRK1 is inhibited, and ZmRFWD3 is released to ubiquitinate O2, facilitating O2 localization into the nucleus. This model links sucrose signaling dynamics with storage protein biosynthesis during grain filling.

**FIGURE 3:**
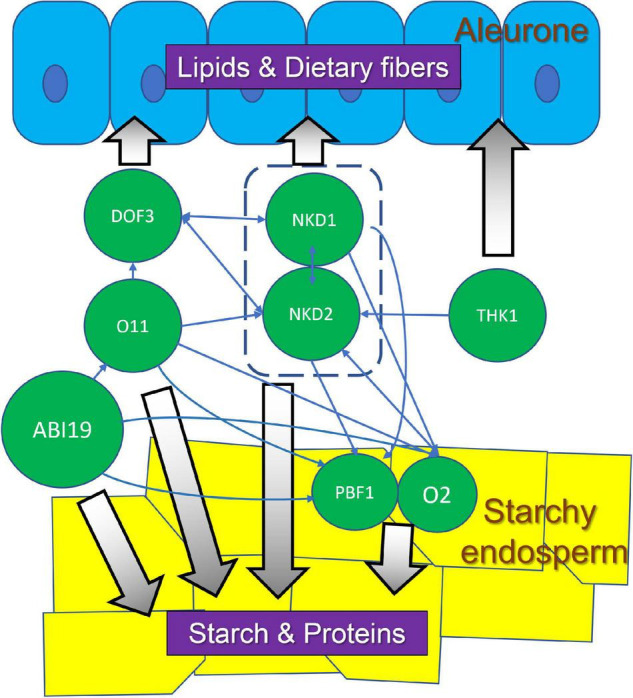
Regulatory interactions among TFs in aleurone and SE, and their potential association with corresponding nutrient compounds. These TFs and associated pathways could be targets for bioengineering to optimize maize seed quality.

A striking point is that many of the known regulators function in multiple cell types and despite advances in our understanding of gene networks, it is still unclear at the GRN level what determines the different cell fate decisions during endosperm development. O2 and PBF are well known TFs important in SE (Zhang et al., 2015; Gontarek et al., 2016) but the *o2* gene is expressed in maize aleurone (Zhan et al., 2018) and a double knockdown of the rice *o2* and *pbf* homologs, *RICE SEED b-ZIPPER 1* (*RISBZ1*) and *RICE PROLAMIN BOX BINDING FACTOR* (*RPBF*), caused multiple layers of disordered aleurone (Kawakatsu et al., 2009). The *o11* mutant, with striking effects on SE size and decreased starch and protein accumulation, also shows multiple layers of irregular aleurone cells (Feng et al., 2018). Similar phenotypes were also observed upon RNA interference of maize *dof3*, which also caused decreased starch accumulation as well as aleurone irregularities (Qi et al., 2017). Conversely, the *nkd* mutant was first recognized for its effect on aleurone development but further analysis showed the *nkd* genes also regulate key functions in the SE (Zhang et al., 2015; Gontarek et al., 2016). Thus, there are complex networks of regulatory interactions amongst TF genes that control both SE and aleurone development (Figure 3). The specific features of these networks that lead to the different cell identities during development remain elusive at this time.

## Endosperm Development and Applications to Improve Grain Quality

“Grain quality” encompasses a variety of traits that vary depending on the specific end use of the grain. They include traits such as grain size, composition, hardness, nutritional value, pathogen resistance, and so on. Knowledge on the regulation of grain development can be used to improve grain properties in two basic ways, either to alter metabolic pathways within the existing cellular context or to alter cellular development. Both cellular development and metabolic pathways were altered during the domestication process without the benefit of understanding the underlying biology. In modern times, the latter approach has received much more attention with significant efforts to understand and manipulate the amount and properties of stored starch, protein and lipids. However, altering cellular development may offer the potential to manipulate grain properties in other ways. Examples of key genes that may be of value for grain improvement are listed in Table 2.

**TABLE 2:** Examples of key genes associated with maize kernel quality.

Gene	Gene model	Functional description	How is it associated with kernel quality?
*O2*	Zm00001d018971	BZIP family transcription factor	High lysine content
*ZP27*	Zm00001d020592	γ-zein protein	Potential *o2*-modifier, improved kernel hardness and high lysine content
*DGAT1-2*	Zm00001d036982	Acyl-CoA:diacylglycerol acyltransferase	High oil content
*WRINKLED1*	Zm00001d005016	HAP3 subunit of the CCAAT-binding transcription factor	High oil content Low starch content
*PBF1*	Zm00001d005100	Prolamin-box binding factor	Affect storage protein content
*MADS47*	Zm00001d046053	MADS-box transcription factor	Affect storage protein content
*BZIP22*	Zm00001d021191	BZIP family transcription factor	Affect storage protein content
*NAC128*	Zm00001d040189	NAC family transcription factor	Affect storage protein and starch fine structure
*NAC130*	Zm00001d008403	NAC family transcription factor	Affect storage protein and starch fine structure
*NKD1*	Zm00001d002654	IDD family zinc finger transcription factor	Affect content of oil, fiber and vitamin in aleurone
*NKD2*	Zm00001d026113	IDD family zinc finger transcription factor	Affect content of oil, fiber and vitamin in aleurone
*THK1*	Zm00001d027278	Scaffolding protein of CCR4-NOT complex	Affect content of oil, fiber and vitamin in aleurone

### Maize Domestication Altered Grain Development

Maize was domesticated around 9,000 years ago from *Zea mays* ssp. *parviglumis*, a grassy teosinte plant native to Mesoamerica (Piperno et al., 2009). Maize and teosinte have striking morphological differences, including kernel characteristics. As reviewed by Flint-Garcia (2017), teosinte grains are small and encased in a hard fruitcase, whereas, modern maize grains are naked and typically about 10 times larger than teosinte. Increased grain size is accompanied by changes in composition, notably an increase in starch content. Elimination of the fruitcase was a critical step in domestication, making the collection and processing of grains for food much easier. Elimination of the fruitcase also removed a physical restriction, which allowed for the dramatic increase in grain size. Modification of this trait was accomplished by mutations and selection of the *teosinte glume architecture1* (*tga1*) gene (Wang et al., 2015). Signatures of selection are also found in the starch biosynthesis genes, *su1, ae1*, and *bt2* (Whitt et al., 2002). For other grain traits, the genetic basis is less clear. Signatures of selection are found in over 1,000 genes indicating that domestication involved the accumulation of many small effects (Wright et al., 2005).

### Grain Improvement Based on Regulation of Metabolic Pathways

There are myriad metabolites in the endosperm that contribute to kernel quality and it is beyond the scope of a single review to cover them all. Here we will mention notable examples for the major classes of storage compounds: starch, protein and oils.

#### Starch

Starch is a deceptively complex molecule and structural variations can lead to variation in quality properties such as gelling temperature and digestibility. Starch metabolism is an example of a system where simple genetic changes can have profound impacts on grain quality and end use. Three examples will be mentioned here:

*Sweet corn* is the most familiar example of altered starch impacting grain characteristics. Mutations in enzymes of the starch biosynthetic pathway impede the incorporation of glucose subunits into starch and thereby cause an accumulation of free sugars in the endosperm. Such grain would not be suitable as feed or most other uses but is valued for human consumption. The *sugary1* (*su1*), *shrunken2* (*sh2*) and *brittle1* (*bt1*) genes are examples of loci where single gene mutations can confer the sweet corn character and form the basis of cultivar breeding programs (Lertrat and Pulam, 2007).

*Waxy* starch is deficient in amylose content. Normal maize endosperm starch is a mixture of amylopectin and amylose in about a 75%:25% ratio. The amylose content influences many starch properties such as gelatinization temperature, pasting and retrogradation, all important for cooking, sizing and other applications (Jane, 2004). Amylose is produced by the granule-bound starch synthase I (GBSSI) protein, encoded by the *waxy1* (*wx1*) gene. Loss-of-function *wx1* mutations produce “waxy” starch nearly devoid of amylose, whereas GWAS identified an allele of *wx1* associated with high amylose content (Li et al., 2018).

*ADPglucose pyrophosphorylase* (*AGPase*) is a rate limiting enzyme that catalyzes the first committed step in starch biosynthesis. Overexpression or enhanced thermostability of the AGPase enzyme can lead to increased starch production and grain yield (Li et al., 2011; Hannah et al., 2017). Interestingly, overexpression increased yield through increased seed size, while the thermostable variant increased yield through increased seed number.

#### Storage Proteins and Quality Protein Maize

Normal maize zeins are characterized by low content of several essential amino acids, most notably lysine, which limits the nutritional value of maize grain in human diets or livestock feed. Mutants of *o2* cause decreased α-zein content accompanied by elevated lysine levels (Mertz et al., 1964). Unfortunately, the improved nutritional composition is associated with unfavorable grain characteristics, including soft and chalky kernel texture, increased susceptibility to insects or fungi, low yields, and unappealing flour characteristics (Villegas, 1994; Habben and Larkins, 1995). To overcome these issues, breeders identified modifier loci that improve grain characteristics while maintaining high lysine content. Such modified *o2* lines are called Quality Protein Maize (QPM) (Prasanna et al., 2001).

Several genes involved in QPM have been identified through QTL mapping. A gene encoding 27 kD γ-zein was linked to the kernel texture phenotype of QPM and RNAi knockdown and genome-wide deletion of γ-zein genes resulted in an opaque and soft kernel phenotypes suggesting that soft kernel texture caused by α-zein deficiency could be compensated by γ-zeins (Lopes et al., 1995; Wu et al., 2010; Yuan et al., 2014). Another QTL mapped to *wx1* (BABU et al., 2015). This is consistent with a proteomic study that found elevated GBSSI activity and altered starch structure in QPM endosperm (Gibbon et al., 2003). These studies suggest that kernel texture derives from complex interactions among proteins, starches and possibly other molecules, and that deficiencies in one molecule can sometimes be compensated by another. These and other QTLs have facilitated marker assisted breeding programs for improved amino acid balance (Gupta et al., 2013; Hossain et al., 2018). Continued study might provide insights for additional strategies to produce improved protein maize lines.

#### Improving Oil Content

Maize oil (corn oil) is mainly used in cooking. Its high smoking point and low saturated fatty acid content make it favorable for frying and human consumption. Also, it may help reduce cholesterol absorption, which could increase the percentage of beneficial high-density lipoproteins (HDL) in blood (Singh et al., 2013). Corn oil is primarily derived from the embryo and the primary determinant of oil percentage is the weight ratio of embryo to endosperm (Yang et al., 2010). Within the endosperm, aleurone cells have the highest oil content. Oil quality is determined by the relative amounts of various fatty acids with different physical characteristics and flavors. Oil quantity (yield) and composition are both of interest for grain improvement. As such, kernel oil content is a complex trait influenced by many factors (Yang et al., 2012; Fang et al., 2021).

QTL-mapping studies identified numerous loci for kernel oil concentration and fatty acid composition. Significantly, many are enzymes involved in the oil metabolic pathway (Yang et al., 2012; Li et al., 2013; Fang et al., 2021). These represent targets for marker assisted breeding or metabolic engineering, and several of them have been utilized to enhance oil content.

A high-oil QTL (qHO6) was associated with an 18.7% increase in oil concentration (in the embryo) as well as altered oil composition with 61.3% more oleic acid and 24.4% less linoleic acid (Zheng et al., 2008). This QTL mapped to a gene encoding acyl-CoA: diacylglycerol acyltransferase (DGAT1-2), a rate limiting enzyme in triacylglycerol synthesis. Transgenic expression of a “normal-oil” *DGAT1-2* allele increased embryo grain oil by 9.3%, while transgenics containing the “high-oil” allele with a phenylalanine inserted at position 469 dramatically increased oil content by 27.9% (Zheng et al., 2008).

Key TFs could also be promising targets for oil improvement. The maize *wrinkled1* gene encodes a HAP3 subunit of the CCAAT-binding factor, and regulates carbon flux between starch and oil biosynthesis during kernel development. Overexpression increased kernel oil content by up to 46% and decreased starch content by approximately 60% (Shen et al., 2010). As knowledge of the molecular basis of oil production accumulates, it is reasonable to expect new strategies to improve maize kernel oil content.

### Grain Improvement Based on Regulation of Cellular Development

Different cell types have varying functions and biochemical compositions. As such, altering the cellular content of a grain has the potential to substantially impact grain characteristics. By and large, this strategy has not been extensively pursued. Here we consider 2 cell types with considerable potential for impacting grain quality, BETL and aleurone.

#### Basal Endosperm Transfer Layer

As the cell type responsible for transporting metabolites from maternal tissues into the endosperm for incorporation into storage products, virtually all grain yield depends on BETL function. As described earlier, expanded expression of *MEG1* caused expanded differentiation of the BETL and resulted in larger kernels (Costa et al., 2012). BETL cells contain an assortment of transporters, including sugars, amino acids, ions and hormones (Thiel, 2014), expressed as part of a BETL gene co-expression module (Zhan et al., 2015). Sugar transport is perhaps the most critical function of the BETL (Cheng et al., 1996; Kang et al., 2009; Sosso et al., 2015). Sugar translocation during grain filling has been subject to selection pressure during domestication. The *sweet4c* gene shows signatures of selection (Sosso et al., 2015). Furthermore, *mn1* is more highly expressed in maize than in teosinte, and in rice, the *mn1* homolog underwent selection during domestication and produced increased grain size and yield upon transgenic overexpression (Wang et al., 2008).

These results support the potential for improving grain size or composition by manipulating BETL development or function. Expanding BETL formation could increase overall solute import and enhance kernel size. Modulating the relative expression levels of various classes of transporters could enhance grain filling or shift grain composition. More complete understanding of the MRP1 transcriptional network will be instrumental in achieving these goals (Dai et al., 2021).

#### Aleurone

As reviewed, the aleurone has many important properties including storage compound remobilization during germination, dietary benefits, mineral storage and pathogen defense (Becraft and Yi, 2011; Gontarek and Becraft, 2017). Commercial maize has a single layer of aleurone and increasing the number of layers could potentially improve certain grain characteristics. Mutants such as *thk1* (Yi et al., 2011) and the multiple aleurone layer (MAL) trait present in the Coroico landrace (Wolf et al., 1972) suggest it should be possible to develop maize cultivars with multiple layers. Transcriptomic analysis of *thk1* endosperm indicated elevated levels of gene expression for pathways associated with aleurone cells, including lipid metabolism, starch degradation, cell wall formation (Wu et al., 2020). Thus, desirable compounds of normal aleurone are likely present at elevated levels in the mutant raising the possibility for enhanced dietary value. The multiple layers of aleurone in barley contributes to the high level of amylase that catalyzes the rapid conversion of starch to fermentable sugars during the malting process. Multiple aleurone layers might potentially lead to new uses for maize grains in malting.

Conversely, eliminating aleurone may be advantageous in certain situations. White rice has the lipid-rich aleurone layer milled off to prevent the grain from going rancid under storage. Similar benefit could potentially be realized from aleurone-free maize grains. Also, phytic acid in aleurone is a major source of phosphate pollution associated with manure runoff which could potentially be alleviated by aleurone-free grain. Such kernels are conferred by the *dek1* mutant (Becraft et al., 2002; Lid et al., 2002).

Existing single gene mutants with the desired aleurone traits combine unfavorable kernel characteristics, including embryo and endosperm defects. To be of practical value, these undesirable effects must be uncoupled from the target traits, similar to what was accomplished with QPM. Further studies on the regulation of endosperm gene expression and GRNs may help facilitate this goal.

### Perspectives

This review summarized studies of maize endosperm development at tissue, cellular and molecular levels, discussed how maize seed development was influenced by domestication, and introduced some current and potential applications to improve maize seed quality based on this knowledge. For future directions, some of the key regulators influencing endosperm development summarized in this article (Table 2) could potentially be utilized for breeding to improve seed quality. Recently, gene network analyses have been applied to maize endosperm development studies (Zhan et al., 2015), providing a powerful tool to predict central regulators of gene expression modules. These regulators could play important roles in biological processes or metabolic pathways of interest, or could act as modifiers or co-factors to interact with known regulators. Based on the predicted information and modern genetic approaches, we may enhance target phenotypes or suppress undesirable side effects more efficiently than conventional breeding approaches.

## Author Contributions

All authors listed have made a substantial, direct, and intellectual contribution to the work, and approved it for publication.

## Conflict of Interest

The authors declare that the research was conducted in the absence of any commercial or financial relationships that could be construed as a potential conflict of interest.

## Publisher’s Note

All claims expressed in this article are solely those of the authors and do not necessarily represent those of their affiliated organizations, or those of the publisher, the editors and the reviewers. Any product that may be evaluated in this article, or claim that may be made by its manufacturer, is not guaranteed or endorsed by the publisher.

## References

[B1] Abendroth L. J. Elmore R. W. Boyer M. J. Marlay S. K. (2011). *Corn Growth and Development.* Ames, IA: Iowa State University Cooperative Extension Service.

[B2] Arcalis E. Stadlmann J. Marcel S. Drakakaki G. Winter V. Rodriguez J. (2010). The changing fate of a secretory glycoprotein in developing maize endosperm. *Plant Physiol.* 153 693–702. 10.1104/pp.109.152363 20388665 PMC2879800

[B3] BABU B. K. Agrawal P. K. Saha S. Gupta H. S. (2015). Mapping QTLs for opaque2 modifiers influencing the tryptophan content in quality protein maize using genomic and candidate gene-based SSRs of lysine and tryptophan metabolic pathway. *Plant Cell Rep.* 34 37–45. 10.1007/s00299-014-1685-5 25236159

[B4] Barrero C. Muniz L. M. Gomez E. Hueros G. Royo J. (2006). Molecular dissection of the interaction between the transcriptional activator ZmMRP-1 and the promoter of BETL-1. *Plant Mol. Biol.* 62 655–668. 10.1007/s11103-006-9047-5 17001496

[B5] Barrero C. Royo J. Grijota-Martinez C. Faye C. Paul W. Sanz S. (2009). The promoter of ZmMRP-1, a maize transfer cell-specific transcriptional activator, is induced at solute exchange surfaces and responds to transport demands. *Planta* 229 235–247. 10.1007/s00425-008-0823-0 18830706 PMC2757625

[B6] Becraft P. W. Asuncion-Crabb Y. T. (2000). Positional cues specify and maintain aleurone cell fate in maize endosperm development. *Development* 127 4039–4048. 10.1242/dev.127.18.4039 10952901

[B7] Becraft P. W. Yi G. (2011). Regulation of aleurone development in cereal grains. *J. Exp. Bot.* 62 1669–1675. 10.1093/jxb/erq372 21109580

[B8] Becraft P. W. Brown R. C. Lemmon B. E. Opsahl-Ferstad H. G. Olsen O.-A. (2001). “Endosperm development,” in *Current Trends In The Embryology Of Angiosperms*, ed. Bhojwani S. S. (Dordrecht: Kluwer), 353–374. 10.1007/978-94-017-1203-3_14

[B9] Becraft P. W. Li K. Dey N. Asuncion-Crabb Y. T. (2002). The maize *dek1* gene functions in embryonic pattern formation and in cell fate specification. *Development* 129 5217–5225. 10.1242/dev.129.22.5217 12399313

[B10] Becraft P. W. Stinard P. S. Mccarty D. R. (1996). CRINKLY4: a TNFR-like receptor kinase involved in maize epidermal differentiation. *Science (New York, N.Y.)* 273 1406–1409. 10.1126/science.273.5280.1406 8703079

[B11] Bhave M. R. Lawrence S. Barton C. Hannah C. (1990). ldentification and molecular characterization of *shrunken*-2 cDNA clones of maize. *Plant Cell* 2 581–588. 10.1105/tpc.2.6.581 1967077 PMC159913

[B12] Bonello J. F. Opsahl-Ferstad H. G. Perez P. Dumas C. Rogowsky P. M. (2000). *Esr* genes show different levels of expression in the same region of maize endosperm. *Gene* 246 219–227. 10.1016/s0378-1119(00)00088-3 10767543

[B13] Brink R. A. Cooper D. C. (1947). Effect of the *De17* allele on development of the maize caryopsis. *Genetics* 32 350–368. 10.1093/genetics/32.4.350 17247249 PMC1209383

[B14] Brown R. C. Lemmon B. E. (2007). “The developmental biology of cereal endosperm,” in *Endosperm, Developmental, and Molecular Biology* ed. Olsen O. A. (Berlin: Springer-Verlag).

[B15] Brown R. C. Lemmon B. E. Olsen O. A. (1996). Polarization predicts the pattern of cellularization in cereal endosperm. *Protoplasma* 192 168–177. 10.1007/bf01273889

[B16] Brugiere N. Humbert S. Rizzo N. Bohn J. Habben J. E. (2008). A member of the maize isopentenyl transferase gene family, Zea mays isopentenyl transferase 2 (ZmIPT2), encodes a cytokinin biosynthetic enzyme expressed during kernel development. Cytokinin biosynthesis in maize. *Plant Mol. Biol.* 67 215–229. 10.1007/s11103-008-9312-x 18311542

[B17] Burton R. A. Fincher G. B. (2014). Evolution and development of cell walls in cereal grains. *Front. Plant Sci.* 5:456. 10.3389/fpls.2014.00456 25309555 PMC4161051

[B18] Cai G. Faleri C. Del Casino C. Hueros G. Thompson R. Cresti M. (2002). Subcellular localisation of BETL-1, -2 and -4 in Zea mays L. *endosperm*. *Sexual Plant Reproduction* 15 85–98. 10.1007/s00497-002-0141-9

[B19] Charlton W. L. Keen C. L. Merriman C. Lynch P. Greenland A. J. Dickinson H. G. (1995). Endosperm development in Zea mays implication of gametic imprinting and paternal excess in regulation of transfer layer development. *Development* 121 3089–3097. 10.1242/dev.121.9.3089

[B20] Chen J. Y. Lausser A. Dresselhaus T. (2014). Hormonal responses during early embryogenesis in maize. *Biochem. Soc. Trans.* 42 325–331. 10.1042/BST20130260 24646239

[B21] Cheng W. H. Taliercio E. W. Chourey P. S. (1996). The *miniature1* seed locus of maize encodes a cell wall invertase required for normal development of endosperm and maternal cells in the pedicel. *Plant Cell* 8 971–983. 10.1105/tpc.8.6.971 12239408 PMC161152

[B22] Chourey P. S. Hueros G. (2017). “The basal endosperm transfer layer (BETL): gateway to the maize kernel,” in *Maize Kernel Development*, ed. Larkins B. A. (Wallingford: CABI Intl), 56–67. 10.1079/9781786391216.0056

[B23] Coelho C. M. Dante R. A. Sabelli P. A. Sun Y. Dilkes B. P. Gordon-Kamm W. J. (2005). Cyclin-dependent kinase inhibitors in maize endosperm and their potential role in endoreduplication. *Plant Physiol.* 138 2323–2336. 10.1104/pp.105.063917 16055680 PMC1183418

[B24] Colasanti J. Tremblay R. Wong A. Y. Coneva V. Kozaki A. Mable B. K. (2006). The maize INDETERMINATE1 flowering time regulator defines a highly conserved zinc finger protein family in higher plants. *BMC Genomics* 7:158. 10.1186/1471-2164-7-158 16784536 PMC1586020

[B25] Cooper D. C. (1951). Caryopsis development following matings between diploid and tetraploid strains of Zea mays. *Am. J. Botany* 38 702–708.

[B26] Cossegal M. Vernoud V. Depege N. Rogowsky P. M. (2007). “The embryo surrounding region,” in *Endosperm, Developmental, and Molecular Biology* ed. Olsen O. A. (Berlin: Springer-Verlag).

[B27] Costa L. M. Yuan J. Rouster J. Paul W. Dickinson H. Gutierrez-Marcos J. F. (2012). Maternal control of nutrient allocation in plant seeds by genomic imprinting. *Curr. Biol.* 22 160–165. 10.1016/j.cub.2011.11.059 22245001

[B28] Dai D. Ma Z. Song R. (2021). Maize endosperm development. *J. Integr. Plant Biol.* 63 613–627. 10.1111/jipb.13069 33448626

[B29] Dante R. A. Larkins B. A. Sabelli P. A. (2014). Cell cycle control and seed development. *Front. Plant Sci.* 5:493. 10.3389/fpls.2014.00493 25295050 PMC4171995

[B30] Davis R. W. Smith J. D. Cobb B. G. (1990). A Light and electron-microscope investigation of the transfer cell region of maize caryopses. *Canadian J. Botany-Revue Canadienne De Botanique* 68 471–479. 10.1139/b90-063

[B31] Doll N. M. Just J. Brunaud V. Caius J. Grimault A. Depege-Fargeix N. (2020). Transcriptomics at maize embryo/endosperm interfaces identifies a transcriptionally distinct endosperm subdomain adjacent to the embryo scutellum. *Plant Cell* 32 833–852. 10.1105/tpc.19.00756 32086366 PMC7145466

[B32] Dominguez F. Cejudo F. J. (2014). Programmed cell death (PCD): an essential process of cereal seed development and germination. *Front. Plant Sci.* 5:366. 10.3389/fpls.2014.00366 25120551 PMC4112785

[B33] Duvick D. N. (1961). Protein granules of maize endosperm cells. *Cereal Chem.* 38 374–385.

[B34] Egli D. B. (2006). The role of seed in the determination of yield of grain crops. *Australian J. Agricultural Res.* 57 1237–1247. 10.1071/ar06133

[B35] Fang H. Fu X. Ge H. Zhang A. Shan T. Wang Y. (2021). Genetic basis of maize kernel oil-related traits revealed by high-density SNP markers in a recombinant inbred line population. *BMC Plant Biol.* 21:344. 10.1186/s12870-021-03089-0 34289812 PMC8293480

[B36] Feng F. Qi W. Lv Y. Yan S. Xu L. Yang W. (2018). OPAQUE11 is a central hub of the regulatory network for maize endosperm development and nutrient metabolism. *Plant Cell* 30 375–396. 10.1105/tpc.17.00616 29436476 PMC5868688

[B37] Flint-Garcia S. A. (2017). “Kernel evolution: from teosinte to maize,” in *Maize kernel development*, ed. Larkins B. A. (Wallingford: CABI). 10.1079/9781786391216.0001

[B38] Gao R. Dong S. Fan J. Hu C. (1998). Relationship between development of endosperm transfer cells and grain mass in maize. *Biol. Plant.* 41 539–546. 10.1023/A:1001840316163

[B39] Gibbon B. C. Wang X. Larkins B. A. (2003). Altered starch structure is associated with endosperm modification in quality protein maize. *Proc. Natl. Acad. Sci. U S A.* 100 15329–15334. 10.1073/pnas.2136854100 14660797 PMC307567

[B40] Gómez E. Royo J. Guo Y. Thompson R. Hueros G. (2002). Establishment of cereal endosperm expression domains: identification and properties of a maize transfer cell-specific transcription factor, ZmMRP-1. *Plant Cell* 14 599–610. 10.1105/tpc.010365 11910007 PMC150582

[B41] Gómez E. Royo J. Muñiz L. M. Sellam O. Paul W. Gerentes D. (2009). The maize transcription factor myb-related protein-1 is a key regulator of the differentiation of transfer cells. *Plant Cell* 21 2022–2035. 10.1105/tpc.108.065409 19574436 PMC2729601

[B42] Gontarek B. C. Becraft P. W. (2017). “Aleurone,” in *Maize Kernel Development*, ed. Larkins B. A. (Boston: CABI), 68–80. 10.1079/9781786391216.0068

[B43] Gontarek B. C. Neelakandan A. K. Wu H. Becraft P. W. (2016). NKD transcription factors are central regulators of maize endosperm development. *Plant Cell* 28 2916–2936. 10.1105/tpc.16.00609 27895224 PMC5240740

[B44] Grafi G. Larkins B. A. (1995). Endoreduplication in Maize endosperm: involvement of m phase-promoting factor inhibition and induction of s phase-related kinases. *Science* 269 1262–1264. 10.1126/science.269.5228.1262 17732113

[B45] Gruis D. Guo H. N. Selinger D. Tian Q. Olsen O. A. (2006). Surface position, not signaling from surrounding maternal tissues, specifies aleurone epidermal cell fate in maize. *Plant Physiol.* 141 898–909. 10.1104/pp.106.080945 16698897 PMC1489889

[B46] Guo X. M. Yuan L. L. Chen H. Sato S. J. Clemente T. E. Holding D. R. (2013). Nonredundant function of zeins and their correct stoichiometric ratio drive protein body formation in maize endosperm. *Plant Physiol.* 162 1359–1369. 10.1104/pp.113.218941 23677936 PMC3707540

[B47] Gupta H. S. Raman B. Agrawal P. K. Mahajan V. Hossain F. Thirunavukkarasu N. (2013). Accelerated development of quality protein maize hybrid through marker-assisted introgression of *opaque-2* allele. *Plant Breed.* 132 77–82.

[B48] Gutierrez-Marcos J. F. Costa L. M. Evans M. M. S. (2006). maternal gametophytic *baseless1* is required for development of the central cell and early endosperm patterning in Maize (Zea mays). *Genetics* 174 317–329. 10.1534/genetics.106.059709 16849604 PMC1569813

[B49] Gutiérrez-Marcos J. F. Costa L. M. Biderre-Petit C. Khbaya B. O’sullivan D. M. Wormald M. (2004). *m**a**t**e**r**n**a**l**l**y**e**x**p**r**e**s**s**e**d**g**e**n**e*1 is a novel maize endosperm transfer cell-specific gene with a maternal parent-of-origin pattern of expression. *Plant Cell* 16 1288–1301. 10.1105/tpc.019778 15105441 PMC423216

[B50] Habben J. E. Larkins B. A. (1995). “Improving protein quality in seeds,” in *Seed Development and Germination*, eds Kijel J. Galili G. (New York, NY: Marcel Dekker, Inc.).

[B51] Hannah L. C. (2007). “Starch formation in the cereal endosperm,” in *Endosperm: Developmental and Molecular Biology*, ed. Olsen O. A. (Berlin: Springer-Verlag).

[B52] Hannah L. C. Shaw J. R. Clancy M. A. Georgelis N. Boehlein S. K. (2017). A *brittle-2* transgene increases maize yield by acting in maternal tissues to increase seed number. *Plant Direct* 1 1–9. 10.1002/pld3.29 31245677 PMC6508519

[B53] He Y. Yang Q. Yang J. Wang Y. F. Sun X. Wang S. (2021). *shrunken4* is a mutant allele of ZmYSL2 that affects aleurone development and starch synthesis in maize. *Genetics* 218:iyab070. 10.1093/genetics/iyab070 34009311 PMC8225342

[B54] Holding D. R. (2014). Recent advances in the study of prolamin storage protein organization and function. *Front. Plant Sci.* 5:276. 10.3389/fpls.2014.00276 24999346 PMC4064455

[B55] Hossain F. Muthusamy V. Pandey N. Vishwakarma A. K. Baveja A. Zunjare R. U. (2018). Marker-assisted introgression of *opaque2* allele for rapid conversion of elite hybrids into quality protein maize. *J. Genet.* 97 287–298. 10.1007/s12041-018-0914-z 29666347

[B56] Hu M. Zhao H. Yang B. Yang S. Liu H. Tian H. (2021). ZmCTLP1 is required for the maintenance of lipid homeostasis and the basal endosperm transfer layer in maize kernels. *New Phytol.* 232 2384–2399. 10.1111/nph.17754 34559890 PMC9292782

[B57] Hueros G. Royo J. Maitz M. Salamini F. Thompson R. D. (1999). Evidence for factors regulating transfer cell-specific expression in maize endosperm. *Plant Mol. Biol.* 41 403–414. 10.1023/a:1006331707605 10598106

[B58] Jane J.-L. (2004). “Starch: structure and properties,” in *J Chemical Functional Properties of Food Saccharides*, ed. Tomasik P. (Boca Raton, FL: CRC Press), 82–96.

[B59] Ji C. Xu L. Li Y. Fu Y. Li S. Wang Q. (2021). The O2-ZmGRAS11 transcriptional regulatory network orchestrates the coordination of endosperm cell expansion and grain filling in maize. *Mol. Plant.* In press. 10.1016/j.molp.2021.11.013 34848346

[B60] Jia M. Wu H. Clay K. L. Jung R. Larkins B. A. Gibbon B. C. (2013). Identification and characterization of lysine-rich proteins and starch biosynthesis genes in the *opaque2* mutant by transcriptional and proteomic analysis. *BMC Plant Biol.* 13:60. 10.1186/1471-2229-13-60 23586588 PMC3762070

[B61] Jin P. Guo T. Becraft P. W. (2000). The maize CR4 receptor-like kinase mediates a growth factor-like differentiation response. *Genesis* 27 104–116. 10951503 10.1002/1526-968x(200007)27:3<104::aid-gene30>3.0.co;2-i

[B62] Kang B. H. Xiong Y. Williams D. S. Pozueta-Romero D. Chourey P. S. (2009). *Miniature1*-encoded cell wall invertase is essential for assembly and function of wall-in-growth in the maize endosperm transfer cell. *Plant Physiol.* 151 1366–1376. 10.1104/pp.109.142331 19759348 PMC2773079

[B63] Kawakatsu T. Yamamoto M. P. Touno S. M. Yasuda H. Takaiwa F. (2009). Compensation and interaction between RISBZ1 and RPBF during grain filling in rice. *Plant J.* 59 908–920. 10.1111/j.1365-313X.2009.03925.x 19473328

[B64] Khoo U. Wolf M. J. (1970). Origin and development of protein granules in maize endosperm. *Am. J. Botany* 57 1042–1050. 10.1002/j.1537-2197.1970.tb09907.x

[B65] Kiesselbach T. A. (1949). The structure and reproduction of corn. *Univ. Nebraska Agric. Stat. Res. Bull.* 161 1–96.

[B66] Kiesselbach T. A. Walker E. R. (1952). Structure of certain specialized tissues in the kernel of corn. *Am. J. Botany* 39 561–569. 10.1002/j.1537-2197.1952.tb13069.x

[B67] Kowles R. V. Phillips R. L. (1985). DNA amplification patterns in maize endosperm nuclei during kernel development. *Proc. Natl. Acad. Sci. U S A.* 82 7010–7014. 10.1073/pnas.82.20.7010 16593620 PMC391299

[B68] Kowles R. V. Phillips R. L. (1988). Endosperm development in maize. *Int. Rev. Cytol.* 112 97–136. 10.1016/S0074-7696(08)62007-0

[B69] Kumar S. B. Venkateswaran K. Kundu S. (2010). Alternative conformational model of a seed protein *DEK1* for better understanding of structure-function relationship. *J. Proteins Proteom.* 1 77–90.

[B70] Kyle D. J. Styles E. D. (1977). Development of aleurone and sub-aleurone-layers in maize. *Planta* 137 185–193. 10.1007/BF00388149 24420652

[B71] Larkins B. A. Dilkes B. P. Dante R. A. Coelho C. M. Woo Y. M. Liu Y. (2001). Investigating the hows and whys of DNA endoreduplication. *J. Exp. Bot.* 52 183–192. 10.1093/jexbot/52.355.183 11283162

[B72] Lending C. R. Larkins B. A. (1989). Changes in the zein composition of protein bodies during maize endosperm development. *Plant Cell* 1 1011–1023. 10.1105/tpc.1.10.1011 2562552 PMC159838

[B73] Leroux B. M. Goodyke A. J. Schumacher K. I. Abbott C. P. Clore A. M. Yadegari R. (2014). Maize early endosperm growth and development: from fertilization through cell type differentiation. *Am. J. Botany* 101 1259–1274. 10.3732/ajb.1400083 25104551

[B74] Lertrat K. Pulam T. (2007). Breeding for increased sweetness in sweet corn. *Int. J. Plant Breeding* 1 27–30.

[B75] Li C. Song R. (2020). The regulation of zein biosynthesis in maize endosperm. *Theor. Appl. Genet.* 133 1443–1453. 10.1007/s00122-019-03520-z 31897513

[B76] Li C. Huang Y. Huang R. Wu Y. Wang W. (2018). The genetic architecture of amylose biosynthesis in maize kernel. *Plant Biotechnol. J.* 16 688–695. 10.1111/pbi.12821 28796926 PMC5787843

[B77] Li C. Qi W. Liang Z. Yang X. Ma Z. Song R. (2020). A SNRK1-ZMRFWD3-OPAQUE2 signaling axis regulates diurnal nitrogen accumulation in maize seeds. *Plant Cell* 32 2823–2841. 10.1105/tpc.20.00352 32699171 PMC7474302

[B78] Li C. Qiao Z. Qi W. Wang Q. Yuan Y. Yang X. (2015). Genome-wide characterization of cis-acting DNA targets reveals the transcriptional regulatory framework of *Opaque2* in maize. *Plant Cell* 27 532–545. 10.1105/tpc.114.134858 25691733 PMC4558662

[B79] Li G. S. Wang D. F. Yang R. L. Logan K. Chen H. Zhang S. S. (2014). Temporal patterns of gene expression in developing maize endosperm identified through transcriptome sequencing. *Proc. Natl. Acad. Sci. U S A.* 111 7582–7587. 10.1073/pnas.1406383111 24821765 PMC4040564

[B80] Li H. Peng Z. Yang X. Wang W. Fu J. Wang J. (2013). Genome-wide association study dissects the genetic architecture of oil biosynthesis in maize kernels. *Nat. Genet.* 45 43–50. 10.1038/ng.2484 23242369

[B81] Li N. Zhang S. Zhao Y. Li B. Zhang J. (2011). Over-expression of AGPase genes enhances seed weight and starch content in transgenic maize. *Planta* 233 241–250. 10.1007/s00425-010-1296-5 20978801

[B82] Lid S. E. Gruis D. Jung R. Lorentzen J. A. Ananiev E. Chamberlin M. (2002). The *d**e**f**e**c**t**i**v**e**k**e**r**n**e**l*1(*d**e**k*1) gene required for aleurone cell development in the endosperm of maize grains encodes a membrane protein of the calpain gene superfamily. *Proc. Natl. Acad. Sci. U S A.* 99 5460–5465. 10.1073/pnas.042098799 11929961 PMC122791

[B83] Lopes M. A. Takasaki K. Bostwick D. E. Helentjaris T. Larkins B. A. (1995). Identification of two opaque2 modifier loci in quality protein maize. *Mol. Gen. Genet.* 247 603–613. 10.1007/BF00290352 7603440

[B84] Lowe J. Nelson O. E. Jr. (1946). Miniature seed-a study in the development of a defective caryopsis in maize. *Genetics* 31 525–533. 10.1093/genetics/31.5.525 17247216 PMC1209347

[B85] McClintock B. (1978). “Development of the maize endosperm as revealed by clones,” in *The Clonal Basis of Development*, eds Subtelny S. Sussex I. M. (New York, NY: Academic Press), 217–237.

[B86] McCurdy D. W. (2015). *Transfer Cells: Novel Cell Types with Unique Wall Ingrowth Architecture Designed for Optimized Nutrient Transport.* Hoboken, NJ: Blackwell-Wiley.

[B87] Mertz E. T. Bates L. S. Nelson O. E. (1964). Mutant gene that changes protein composition and increases lysine content of maize endosperm. *Science* 145 279–280. 10.1126/science.145.3629.279 14171571

[B88] Miller M. E. Chourey P. S. (1992). The maize invertase-deficient *miniature*-1 seed mutation is associated with aberrant pedicel and endosperm development. *Plant Cell* 4 297–305. 10.1105/tpc.4.3.297 12297647 PMC160130

[B89] Mimura M. Kudo T. Wu S. Mccarty D. R. Suzuki M. (2018). Autonomous and non-autonomous functions of the maize *Shohai1* gene, encoding a RWP-RK putative transcription factor, in regulation of embryo and endosperm development. *Plant J.* 95 892–908. 10.1111/tpj.13996 29901832

[B90] Mol R. Matthys-Rochon E. Dumas C. (1994). The kinetics of cytological events during double fertilization in *Zea mays* L. *Plant J.* 5 197–206. 10.1046/j.1365-313X.1994.05020197.x

[B91] Monjardino P. Machado J. Gil F. S. Fernandes R. Salema R. (2007). Structural and ultrastructural characterization of maize coenocyte and endosperm cellularization. *Canadian J. Botany-Revue Canadienne De Botanique* 85 216–223. 10.1139/b06-156

[B92] Monjardino P. Rocha S. Tavares A. C. Fernandes R. Sampaio P. Salema R. (2013). Development of flange and reticulate wall ingrowths in maize (*Zea mays* L.) endosperm transfer cells. *Protoplasma* 250 495–503. 10.1007/s00709-012-0432-4 22814725

[B93] Muniz L. M. Royo J. Gomez E. Barrero C. Bergareche D. Hueros G. (2006). The maize transfer cell-specific type-A response regulator ZmTCRR-1 appears to be involved in intercellular signalling. *Plant J.* 48 17–27. 10.1111/j.1365-313X.2006.02848.x 16925601

[B94] Muniz L. M. Royo J. Gomez E. Baudot G. Paul W. Hueros G. (2010). Atypical response regulators expressed in the maize endosperm transfer cells link canonical two component systems and seed biology. *BMC Plant Biol.* 10:84. 10.1186/1471-2229-10-84 20459670 PMC3017813

[B95] Olsen O. A. (2001). Endosperm development: cellularization and cell fate specification. *Annu. Rev. Plant Physiol. Plant Mol. Biol.* 52 233–267. 10.1146/annurev.arplant.52.1.233 11337398

[B96] Olsen O. A. (2020). The modular control of cereal endosperm development. *Trends Plant Sci.* 25 279–290. 10.1016/j.tplants.2019.12.003 31956036

[B97] Olsen O. A. Becraft P. W. (2013). “Endosperm development,” in *Seed Gemonics*, ed. Becraft P. W. (New York, NY: John Wiley & Sons), 43–63.

[B98] Olsen O.-A. Brown R. C. Lemmon B. E. (1995). Pattern and process of wall formation in developing endosperm. *BioEssays* 17 803–812. 10.1002/bies.950170910

[B99] Opsahl-Ferstad H. G. Ledeunff E. Dumas C. Rogowsky P. M. (1997). ZmEsr, a novel endosperm-specific gene expressed in a restricted region around the maize embryo. *Plant J.* 12 235–246. 10.1046/j.1365-313x.1997.12010235.x 9263463

[B100] Piperno D. R. Ranere A. J. Holst I. Iriarte J. Dickau R. (2009). Starch grain and phytolith evidence for early ninth millennium B.P. maize from the Central Balsas River Valley, Mexico. *Proc. Natl. Acad. Sci. U S A.* 106 5019–5024. 10.1073/pnas.0812525106 19307570 PMC2664021

[B101] Prasanna B. M. Vasal S. K. Kassahun B. Singh N. N. (2001). Quality protein maize. *Curr. Sci.* 81 1308–1319.

[B102] Qi X. Li S. Zhu Y. Zhao Q. Zhu D. Yu J. (2017). ZmDof3, a maize endosperm-specific Dof protein gene, regulates starch accumulation and aleurone development in maize endosperm. *Plant Mol. Biol.* 93 7–20. 10.1007/s11103-016-0543-y 27709320

[B103] Randolph L. F. (1936). Developmental morphology of the caryopsis in maize. *J. Agric. Res.* 53 881–916.

[B104] Reyes F. C. Chung T. Holding D. Jung R. Vierstra R. Otegui M. S. (2011). Delivery of prolamins to the protein storage vacuole in maize aleurone cells. *Plant Cell* 23 769–784. 10.1105/tpc.110.082156 21343414 PMC3077793

[B105] Rocha S. Monjardino P. Mendonca D. Machado A. D. Fernandes R. Sampaio P. (2014). Lignification of developing maize (*Zea mays* L.) endosperm transfer cells and starchy endosperm cells. *Front. Plant Sci.* 5:102. 10.3389/fpls.2014.00102 24688487 PMC3960489

[B106] Rodrigues J. A. Zilberman D. (2015). Evolution and function of genomic imprinting in plants. *Genes Dev.* 29 2517–2531. 10.1101/gad.269902.115 26680300 PMC4699382

[B107] Sabelli P. A. (2012). Replicate and die for your own good: endoreduplication and cell death in the cereal endosperm. *J. Cereal Sci.* 56 9–20. 10.1016/j.jcs.2011.09.006

[B108] Sabelli P. A. Larkins B. A. (2009). The contribution of cell cycle regulation to endosperm development. *Sexual Plant Reproduction* 22 207–219. 10.1007/s00497-009-0105-4 20033442

[B109] Sabelli P. A. Liu Y. Dante R. A. Lizarraga L. E. Nguyen H. N. Brown S. W. (2013). Control of cell proliferation, endoreduplication, cell size, and cell death by the retinoblastoma-related pathway in maize endosperm. *Proc. Natl. Acad. Sci. U S A.* 110 E1827–E1836. 10.1073/pnas.1304903110 23610440 PMC3651506

[B110] Schel J. H. N. Kieft H. Vanlammeren A. A. M. (1984). Interactions between embryo and endosperm during early developmental stages of maize caryopses (*Zea mays*). *Canadian J. Botany-Revue Canadienne De Botanique* 62 2842–2853. 10.1139/b84-379

[B111] Schmidt R. J. Burr F. A. Aukerman M. J. Burr B. (1990). Maize regulatory gene *o**p**a**q**u**e*-2 encodes a protein with a “leucine-zipper” motif that binds to zein DNA. *Proc. Natl. Acad. Sci. U S A.* 87 46–50. 10.1073/pnas.87.1.46 2296602 PMC53196

[B112] Schmidt R. J. Ketudat M. Aukerman M. J. Hoschek G. (1992). Opaque-2 is a transcriptional activator that recognizes a specific target site in 22-kD zein genes. *Plant Cell* 4 689–700. 10.1105/tpc.4.6.689 1392590 PMC160165

[B113] Shen B. Allen W. B. Zheng P. Li C. Glassman K. Ranch J. (2010). Expression of *zmlec1* and *zmwri1* increases seed oil production in maize. *Plant Physiol.* 153 980–987. 10.1104/pp.110.157537 20488892 PMC2899924

[B114] Shen B. Li C. Min Z. Meeley R. B. Tarczynski M. C. Olsen O. A. (2003). *sal1* determines the number of aleurone cell layers in maize endosperm and encodes a class E vacuolar sorting protein. *Proc. Natl. Acad. Sci. U S A.* 100 6552–6557. 10.1073/pnas.0732023100 12750475 PMC164484

[B115] Sheridan W. F. Clark J. K. (2017). “Embryo development,” in *Maize kernel Development*, ed. Larkins B. A. (Oxfordshire: CABI). 10.1079/9781786391216.0081

[B116] Singh N. Vasudev S. Yadava D. K. Chaudhary D. P. Prabhu K. V. (2013). “Oil improvement in maize: potential and prospects,” in *Maize: Nutrition Dynamics and Novel Uses.* (eds) Chuadhary D. P. Kumar S. Langyan S. (New Delhi: Springer-Verlag) 10.1007/978-81-322-1623-0_6

[B117] Sosso D. Luo D. Li Q.-B. Sasse J. Yang J. Gendrot G. (2015). Seed filling in domesticated maize and rice depends on SWEET-mediated hexose transport. *Nat. Genet.* 47 1489–1493. 10.1038/ng.3422 26523777

[B118] Spitzer C. Reyes F. C. Buono R. Sliwinski M. K. Haas T. J. Otegui M. S. (2009). The ESCRT-related CHMP1A and B proteins mediate multivesicular body sorting of auxin carriers in Arabidopsis and are required for plant development. *Plant Cell* 21 749–766. 10.1105/tpc.108.064865 19304934 PMC2671707

[B119] Talbot M. J. Offler C. E. Mccurdy D. W. (2002). Transfer cell wall architecture: a contribution towards understanding localized wall deposition. *Protoplasma* 219 197–209. 10.1007/s007090200021 12099220

[B120] Thiel J. (2014). Development of endosperm transfer cells in barley. *Front. Plant Sci.* 5:108. 10.3389/fpls.2014.00108 24723929 PMC3972472

[B121] Tian Q. Olsen L. Sun B. Lid S. E. Brown R. C. Lemmon B. E. (2007). Subcellular localization and functional domain studies of DEFECTIVE KERNEL1 in maize and *Arabidopsis* suggest a model for aleurone cell fate specification involving CRINKLY4 and SUPERNUMERARY ALEURONE LAYER1. *Plant Cell* 19 3127–3145. 10.1105/tpc.106.048868 17933905 PMC2174714

[B122] To T. Estrabillo E. Wang C. Cicutto L. (2008). Examining intra-rater and inter-rater response agreement: a medical chart abstraction study of a community-based asthma care program. *BMC Med. Res. Methodol.* 8:29. 10.1186/1471-2288-8-29 18471298 PMC2396663

[B123] Tran D. Galletti R. Neumann E. D. Dubois A. Sharif-Naeini R. Geitmann A. (2017). A mechanosensitive Ca2+ channel activity is dependent on the developmental regulator DEK1. *Nat. Commun.* 8:1009. 10.1038/s41467-017-00878-w PMC5647327 29044106

[B124] Vilhar B. Kladnik A. Blejec A. Chourey P. S. Dermastia M. (2002). Cytometrical evidence that the loss of seed weight in the *miniature1* seed mutant of maize is associated with reduced mitotic activity in the developing endosperm. *Plant Physiol.* 129 23–30. 12011334 10.1104/pp.001826 PMC1540223

[B125] Villegas E. (1994). “Factors limiting quality protein maize (QPM) development and utilization,” in *Quality Protein Maize 1964-1994*, eds Larkins B. A. Mertz E. T. (Sete Lahoas: EMBRAPA/CNPMS).

[B126] Wang C. Barry J. K. Min Z. Tordsen G. Rao A. G. Olsen O.-A. (2003). The calpain domain of the maize DEK1 protein contains the conserved catalytic triad and functions as a cysteine proteinase. *J. Biol. Chem.* 278 34467–34474. 12824178 10.1074/jbc.M300745200

[B127] Wang E. Wang J. Zhu X. Hao W. Wang L. Li Q. (2008). Control of rice grain-filling and yield by a gene with a potential signature of domestication. *Nat. Genet.* 40 1370–1374. 18820698 10.1038/ng.220

[B128] Wang H. Studer A. J. Zhao Q. Meeley R. Doebley J. F. (2015). Evidence that the origin of naked kernels during maize domestication was caused by a single amino acid substitution in *tga1*. *Genetics* 200 965–974. 25943393 10.1534/genetics.115.175752 PMC4512555

[B129] Wang Z. Ueda T. Messing J. (1998). Characterization of the maize prolamin box-binding factor-1 (PBF-1) and its role in the developmental regulation of the zein multigene family. *Gene* 223 321–332. 9858759 10.1016/s0378-1119(98)00244-3

[B130] Whitt S. R. Wilson L. M. Tenaillon M. I. Gaut B. S. Buckler E. S. T. (2002). Genetic diversity and selection in the maize starch pathway. *Proc. Natl. Acad. Sci. U S A.* 99 12959–12962. 12244216 10.1073/pnas.202476999 PMC130568

[B131] Wolf M. J. Cutler H. C. Zuber M. S. Khoo U. (1972). Maize with multilayer aleurone of high protein content. *Crop Sci.* 12 440–442.

[B132] Woo Y. M. Hu D. W. N. Larkins B. A. Jung R. (2001). Genomics analysis of genes expressed in maize endosperm identifies novel seed proteins and clarifies patterns of zein gene expression. *Plant Cell* 13 2297–2317. 11595803 10.1105/tpc.010240 PMC139160

[B133] Wright S. I. Bi I. V. Schroeder S. G. Yamasaki M. Doebley J. F. Mcmullen M. D. (2005). The effects of artificial selection on the maize genome. *Science* 308 1310–1314. 15919994 10.1126/science.1107891

[B134] Wu H. Becraft P. W. (2021). Comparative transcriptomics and network analysis define gene coexpression modules that control maize aleurone development and auxin signaling. *Plant Genome* 14:e20126. 10.1002/tpg2.20126 PMC12807064 34323399

[B135] Wu H. Gontarek B. C. Yi G. Beall B. D. Neelakandan A. K. Adhikari B. (2020). The *t**h**i**c**k**a**l**e**u**r**o**n**e*1 gene encodes a NOT1 subunit of the CCR4-NOT complex and regulates cell patterning in endosperm. *Plant Physiol.* 184 960–972. 32737073 10.1104/pp.20.00703 PMC7536710

[B136] Wu Y. Holding D. R. Messing J. (2010). γ-Zeins are essential for endosperm modification in quality protein maize. *Proc. Natl. Acad. Sci. U.S.A.* 107 12810–12815. 10.1073/pnas.1004721107 20615951 PMC2919962

[B137] Yang T. Guo L. Ji C. Wang H. Wang J. Zheng X. (2021). The B3 domain-containing transcription factor ZmABI19 coordinates expression of key factors required for maize seed development and grain filling. *Plant Cell* 33 104–128. 33751093 10.1093/plcell/koaa008 PMC8136913

[B138] Yang X. Guo Y. Yan J. Zhang J. Song T. Rocheford T. (2010). Major and minor QTL and epistasis contribute to fatty acid compositions and oil concentration in high-oil maize. *Theor. Appl. Genet.* 120 665–678. 19856173 10.1007/s00122-009-1184-1

[B139] Yang X. Ma H. Zhang P. Yan J. Guo Y. Song T. (2012). Characterization of QTL for oil content in maize kernel. *Theor. Appl. Genet.* 125 1169–1179. 22669301 10.1007/s00122-012-1903-x

[B140] Yi G. Lauter A. M. Scott M. P. Becraft P. W. (2011). The *t**h**i**c**k**a**l**e**u**r**o**n**e*1 mutant defines a negative regulation of maize aleurone cell fate that functions downstream of defective kernel1. *Plant Physiol.* 156 1826–1836. 21617032 10.1104/pp.111.177725 PMC3149929

[B141] Yi G. Neelakandan A. K. Gontarek B. C. Vollbrecht E. Becraft P. W. (2015). The *n**a**k**e**d**e**n**d**o**s**p**e**r**m* genes encode duplicate INDETERMINATE domain transcription factors required for maize endosperm cell patterning and differentiation. *Plant Physiol.* 167 443–456. 25552497 10.1104/pp.114.251413 PMC4326753

[B142] Young T. E. Gallie D. R. (2000). Regulation of programmed cell death in maize endosperm by abscisic acid. *Plant Mol. Biol.* 42 397–414. 10794539 10.1023/a:1006333103342

[B143] Young T. E. Gallie D. R. Demason D. A. (1997). Ethylene-mediated programmed cell death during maize endosperm development of wild-type and *shrunken2* genotypes. *Plant Physiol.* 115 737–751. 12223841 10.1104/pp.115.2.737 PMC158534

[B144] Yuan L. Dou Y. Kianian S. F. Zhang C. Holding D. R. (2014). Deletion mutagenesis identifies a haploinsufficient role for γ-Zein in opaque2 endosperm modification. *Plant Physiol.* 164 119–130. 10.1104/pp.113.230961 24214534 PMC3875793

[B145] Zhan J. Li G. Ryu C. H. Ma C. Zhang S. Lloyd A. (2018). Opaque-2 regulates a complex gene network associated with cell differentiation and storage functions of maize endosperm. *Plant Cell* 30 2425–2446. 30262552 10.1105/tpc.18.00392 PMC6241275

[B146] Zhan J. Thakare D. Ma C. Lloyd A. Nixon N. M. Arakaki A. M. (2015). RNA sequencing of laser-capture microdissected compartments of the maize kernel identifies regulatory modules associated with endosperm cell differentiation. *Plant Cell* 27 513–531. 25783031 10.1105/tpc.114.135657 PMC4558669

[B147] Zhang Z. Dong J. Ji C. Wu Y. Messing J. (2019). NAC-type transcription factors regulate accumulation of starch and protein in maize seeds. *Proc. Natl. Acad. Sci. U S A.* 116 11223–11228. 31110006 10.1073/pnas.1904995116 PMC6561305

[B148] Zhang Z. Qu J. Li F. Li S. Xu S. Zhang R. (2020). Genome-wide evolutionary characterization and expression analysis of SIAMESE-RELATED family genes in maize. *BMC Evol. Biol.* 20:91. 10.1186/s12862-020-01619-2 32727363 PMC7389639

[B149] Zhang Z. Yang J. Wu Y. (2015). Transcriptional regulation of zein gene expression in maize through the additive and synergistic action of *opaque2*, prolamine-box binding factor, and O2 heterodimerizing proteins. *Plant Cell* 27 1162–1172. 25901087 10.1105/tpc.15.00035 PMC4558697

[B150] Zhang Z. Zheng X. Yang J. Messing J. Wu Y. (2016). Maize endosperm-specific transcription factors O2 and PBF network the regulation of protein and starch synthesis. *Proc. Natl. Acad. Sci. U S A.* 113 10842–10847. 27621432 10.1073/pnas.1613721113 PMC5047157

[B151] Zheng P. Allen W. B. Roesler K. Williams M. E. Zhang S. Li J. (2008). A phenylalanine in DGAT is a key determinant of oil content and composition in maize. *Nat. Genet.* 40 367–372. 18278045 10.1038/ng.85

[B152] Zheng Y. K. Wang Z. (2010). Current opinions on endosperm transfer cells in maize. *Plant Cell Rep.* 29 935–942. 20585949 10.1007/s00299-010-0891-z

[B153] Zheng Y. K. Wang Z. Gu Y. J. (2014). Development and function of caryopsis transport tissues in maize, sorghum and wheat. *Plant Cell Rep.* 33 1023–1031. 24652624 10.1007/s00299-014-1593-8

